# Ecological restoration stimulates environmental outcomes but exacerbates water shortage in the Loess Plateau

**DOI:** 10.7717/peerj.13658

**Published:** 2022-07-08

**Authors:** Mbezele Junior Yannick Ngaba, Yves Uwiragiye, Hongzhi Miao, Zhiqin Li, Jianbin Zhou

**Affiliations:** 1 College of Natural Resources and Environment, Northwest A&F University, Yangling, Shaanxi, China; 2 Ministry of Agriculture, Key Laboratory of Plant Nutrition and the Agri-Environment in Northwest China, Yangling, Shaanxi, China; 3 Crop production, University of Technology and Arts of Byumba, Byumba, Northern, Rwanda, Rwanda

**Keywords:** Ecological restoration, Environmental outcomes, Loess plateau, Grain for green project, Meta-analysis, Water shortage

## Abstract

Restoration is the natural and intervention-assisted set of processes designed to promote and facilitate the recovery of an ecosystem that has been degraded, damaged, or destroyed. However, it can also have an adverse effect on the environment. Thus, assessing an ecological restoration project’s impact is crucial to determining its success and optimum management strategies. We performed a meta-analysis concerning the environmental outcomes during the years 2000–2015 resulting from the “Grain for Green” Project (GFGP) implementation in the Loess Plateau (LP). Data were gathered from 40 peer-reviewed English-language articles chosen from a pool of 332 articles. The results showed that, on average, GFGP increased forest coverage by 35.7% (95% CI [24.15–47.52%]), and grassland by 1.05% (95% CI [0.8–1.28%]). At the same time, GFGP has a positive impact on soil carbon (C) sequestration, net ecosystem production (NEP), and net primary production (NPP), from the years 2000 to 2015 by an average of 36% (95% CI [28.96–43.18%]), 22.7% (95% CI [9.10–36.79%]), and 13.5% (95% CI [9.44–17.354%]), respectively. Soil erosion, sediment load, runoff coefficient, and water yield were reduced by 13.3% (95% CI [0.27–25.76%]), 21.5% (95% CI [1.50–39.99%]), 22.4% (95% CI [5.28–40.45%]) and 43.3% (95% CI [27.03–82.86%]), respectively, from the years 2000 to 2015. Our results indicate that water supply decreased with the increase of vegetation coverage. Therefore, to balance the needs for green space, GFGP policies and strategies should recover, enhance, and sustain more resilient ecosystems.

## Introduction

Forests are critical to human well-being, providing numerous ecosystem services (Costanza et al., 1997). However, in recent years, the rapid advancement in forest management practices and technological improvements have uncovered the synergies and trade-offs associated with different management options (Pukkala, 2016; Raudsepp-Hearne, Peterson & Bennett, 2010). The Chinese Loess Plateau was degraded after thousands of years of agricultural exploitation, and it is one of the most degraded areas in the world. In the last century, this region’s fragmentation and environmental degradation increased due to population growth and soil vulnerability to degradation (Fu et al., 2011). This pressure increased soil degradation, river siltation, reduced food production, and triggered poverty. Forest coverage decreased from nearly 50% 2000 years ago to 33% 1500 years ago and 6.1% in 1949 (Shi, 2001). To improve people’s well-being and restore degraded ecosystems, the Chinese government implemented a large-scale ecological restoration program in 1999: the “Grain for Green” Project (GFGP), funded by the government. This program is one of the most significant reforestation projects conducted globally. It aims to restore and increase vegetation cover, control soil erosion by converting cropland to forest, grasslands, or shrubland, introduce more sustainable land management practices and improve agricultural production in the LP (Chen et al., 2015; Hua et al., 2016; Sun et al., 2015). The GFGP covered 25 provinces and 1,600 counties and involved nearly 15 million households (Zhiyong, 2004). Since its implementation, the Chinese government has invested nearly US$28.8 billion and expects to invest more than US$40 billion by 2050 (Feng et al., 2013; Xu et al., 2004).

In recent years, advanced practices and operations in ecological restoration have multiplied in the LP, which has become the most successful ecological restoration zone in China (Fu et al., 2017). Afforestation has been considered the most effective technique for ecological restoration in the LP because it can mitigate human disturbance and restore ecosystem functions (Chen et al., 2015; Feng et al., 2016). So far, promising results have been observed in the LP, and more is yet to come. Several studies examined the impact of large-scale afforestation (vegetation greening) on environmental outcomes in semi-arid or arid regions (Feng et al., 2016; Li et al., 2019; Li et al., 2018; Wu et al., 2019c). However, evidence on the impacts of afforestation in the LP through GFGP implementation remains controversial.

For instance, many positive environmental outcomes have been identified, such as increased vegetation cover (Jiang, Zhang & Zhang, 2018; Jiao et al., 2012; Xiao, 2014); net primary productivity, C sequestration (Deng et al., 2018; Fang et al., 2017; Feng et al., 2020; Gang et al., 2018; Wu et al., 2019a); improvement of vegetation structure and species diversity (Jiao et al., 2012; Zhao et al., 2018) and soil conservation (Dou et al., 2020; Feng et al., 2020; Fu et al., 2011; Li et al., 2012; Lü et al., 2012; Wu et al., 2019b). At the same time, it can also lead to adverse effects on ES and human beings (Chen et al., 2015; Geng et al., 2020). For instance, a poor understanding of land-use planning, environmental management, and hydrological processes can lead to unmet management targets (McVicar et al., 2007; Yu et al., 2020). Moreover, non-adapted or/and inappropriate practices such as inappropriate grazing, overgrazing, removal of native vegetation, restriction of water flows, and large-scale ecological restoration can have irreversible repercussions on an ecosystem and lead to the failure of a restoration project (*e.g*., water cycle changes, a high mortality rate of planted trees, and food production). Therefore, it is crucial to assess the ecological restoration in the LP by monitoring and evaluating the temporal impact of afforestation on environmental outcomes induced by GFGP.

Besides, although environmental assessment is currently gaining further momentum in the LP, most of those studies focused on one or many biophysical components of the environment: water (Ge et al., 2020; Li et al., 2016; Zhang et al., 2015); or soil (Dou et al., 2020; Feng et al., 2012; Lv et al., 2019) rather than presenting an overview of the revegetation impact on the LP as a whole system. These contradictory results can be explained by the fact that there are connections among ecosystem services because of landscape connectivity. Consequently, any action that aims to restore or ameliorate one or many ecosystem services can cause the degradation of other ecosystem services (Assessment, 2005; Bennett, Peterson & Gordon, 2009). Despite considerable scientific attention to the LP, the overall response to environmental outcomes in the fight against soil erosion remains unclear. We assessed the environmental impact of the GFGP in the LP from 2000 to 2015 using the meta-analysis method to evaluate the impacts of the policies implemented. This study shows how response ratios of environmental outcomes change with the age of restoration from 2000 to 2015 in the LP. Specifically, our objectives were to determine how afforestation affects the responses of (i) vegetation coverage, (ii) soil and water resources, and (iii) soil C fluxes and sequestration over time.

## Materials and Methods

The Loess Plateau (LP) is located in central China, between 33°43′7″–41°16′7″N and 100°54′ 7″–114°33′ 7″E on the upper and middle reaches of the Yellow River (Fig. S1). The Loess Plateau is located in the middle reaches of the Yellow River Basin, which covers an area of 640,000 km^2^ and includes seven administrative provinces (specifically, Gansu, Inner Mongolia, Ningxia, Qinghai, Shaanxi, Shanxi, and Henan). The average annual precipitation ranges from 144 mm in the north to 790 mm in the south, with the majority falling between June and September (Jiang, Zhang & Zhang, 2018). The average annual temperature ranges from 6 to 14 °C. The soil is primarily loess and rich in nutrients, making it suitable for agricultural production and the majority of soil texture ranges from fine silt to silt, making it prone to erosion (Geng et al., 2020).

### Review time horizon

In this study, the period of reviews chosen was 15 years (2000 to 2015) and carried out until December 31st, 2020, in the LP (Fig. S1). The time frame selected is linked to the start-up date of the GFGP implementation and is due to the data availability. This phase can be identified as “revegetation for the environment,” characterized by increased forest coverage and a decrease in cropland (Wu et al., 2020). Figure S2 presents the number of studies published per year, showing a gap from 2000 to 2015 (22.64% of the studies).

### Literature search, selection, and classification of papers

The literature database comprises peer-reviewed articles published in English and selected from different databases such as Semantic Scholar, Science Direct, Google Scholar, and Web of Knowledge (Table S1). All field studies evaluate afforestation’s effects on environmental outcomes in the LP. Keywords for searches included combinations of the project name and location (*e.g*., “Grain for Green”, “Grain to Green Program”, “GFG”, “GFGP”, “GTGP”, “Loess Plateau”, “China”, “GGP”), management practices and ecosystem services (*e.g*., “afforestation”, “impacts”, “effect”, “reforestation”, “soil erosion”, “vegetation restoration”, “carbon sequestration”, “evapotranspiration”, “runoff”, “water yield”, “sediment load”, “net ecosystem production”, “NEP”, “net primary production”, “NPP”, “afforestation”, “land use”, “land use changes”) (Table S2).

The following conditions were considered to select and classify articles: (i) The selected studies were carried out at the Loess plateau scale; (ii) quantitative information on environmental outcomes (forest coverage, grassland, evapotranspiration, albedo, sediment load, water yield, runoff coefficient, soil erosion, C sequestration, NEP and NPP) are directly provided, could be estimated, or extracted from the articles in graphs or tables; (iii) The studies must contain at least one of the target variables, and they were examined in all treatments; (iv) The means, standard errors or deviations, and sample sizes for control and treatment groups can be directly obtained from text and tables or could be obtained from the provided digitized graphs. The review framework was obtained in this study by combining two methods used to select, classify the studies, describe, and analyze the data (Arksey & O’Malley, 2005; Flynn et al., 1990). Out of 332 studies, 40 articles were selected through this screening process (Fig. S3). The articles relevant to this study were published in 24 journals from 2000 to 2020. The number of articles reported in each journal is shown in Fig. S4.

### Mixed-effects meta-regression analysis

We tested the impact of C sequestration, grassland evaporation, forest coverage, albedo, MAT, MAP, soil erosion, runoff, sediment load, water yield, NEP, and NPP on water yield in a model of mixed-effects meta-regression using the *“glmulti”* package in R. A cutoff of 0.8 was set to differentiate between important and unimportant predictors.

### Data and statistical analysis

The study methodology applied has been used in previous works (Bhatia & Gangwani, 2020; Hermundsdottir & Aspelund, 2021). The data was extracted from selected publications directly from the text, tables, and figures using Graph Grabber 2.0.2 software. We e-mailed the authors to request additional data when it was not complete. Spearman correlation was used to assess the relationship between soil erosion, C sequestration, water yield change, and forest coverage. Correlations were considered significant at *p* < 0.05. Statistical analyses and graphs were determined with SPSS 26.0 software (SPSS, Inc., Chicago, IL, USA) and OriginPro 2021.

The effects of afforestation on ecological outcomes were calculated each year by comparing the control result (at year n) with the changes observed (at year n + 1) using the following equation:

(1) $${\rm ln}R={\rm ln}\ ({X_n}/{X_{n + i}}{\rm )}$$

where lnR is the effect size calculated as the natural log of the response ratio (RR) for each comparison, X_*n*_ and X_*n+i*_ are the ecological outcomes effect group (n + *i*) and the control group (n), respectively; *i* represent the group effect observed within 2000 and 2015 (*e.g*., the effect size in the year 2000 will be determined by comparing the outcomes effect group in 2000, which represents the change observed and the outcomes control group in 1999).

The effects of GFGP on ecological outcomes were calculated between 2000 and 2015 by comparing the results of the control (in 2000) with the changes observed (in 2015) using the following equation:

(2) $${\rm ln}R={ ln}\ ({X_t}/{X_c}{\rm )}$$

where lnR is the effect size calculated as the natural log of the response ratio for each comparison, X_*t*_ and *X_c_* are the ecological outcome effect groups (in 2015) and the control group (in 1999), respectively.

The average mean response ratio was calculated from the RR of individual pair comparisons between treatments (age of restoration after 2000) and controls. A variance ( $v$) was calculated as the sample sizes of a subject variable in the treatment and control groups, respectively.

(3) $$v = \displaystyle{{S_t^2} \over {{n_t}\overline {X_t^2} }}\ +\ \displaystyle{{S_c^2} \over {{n_c}\overline {X_c^2} }},$$

where ${n_t}$ and ${n_c}$ are sample sizes of the concerned variable in the treatment and control groups, respectively. We calculate the mean effect size and variance using a weighted random-effects approach. Mean effect sizes and 95% confidence intervals were generated by bootstrapping (4,999 iterations). The effect of GFGP was considered significant if the 95% confidence intervals (CI) did not overlap with zero. For literature sources where the standard error (SE) rather than the standard deviation (SD) was reported, we recalculated the SD by:

(4) $${\rm SD = SE\ \times\ }\sqrt {\rm N},$$

where N is the number of replications, figures were drawn using the OriginPro software version 8.5. The meta-analysis procedures were conducted using MetaWin 2.1 software (Sinauer Associates, Inc., Sunderland, MA, USA). Values of lnR were transformed into percentage changes by:

(5) $${\rm Effect \; size}\left( {\rm \% } \right) = \left( {{\rm exp}\left( {{\rm ln}R} \right)-1} \right) \times {\rm 100\% }$$

## Results

### Effect of GFGP on vegetation coverage

**Figure 1:**
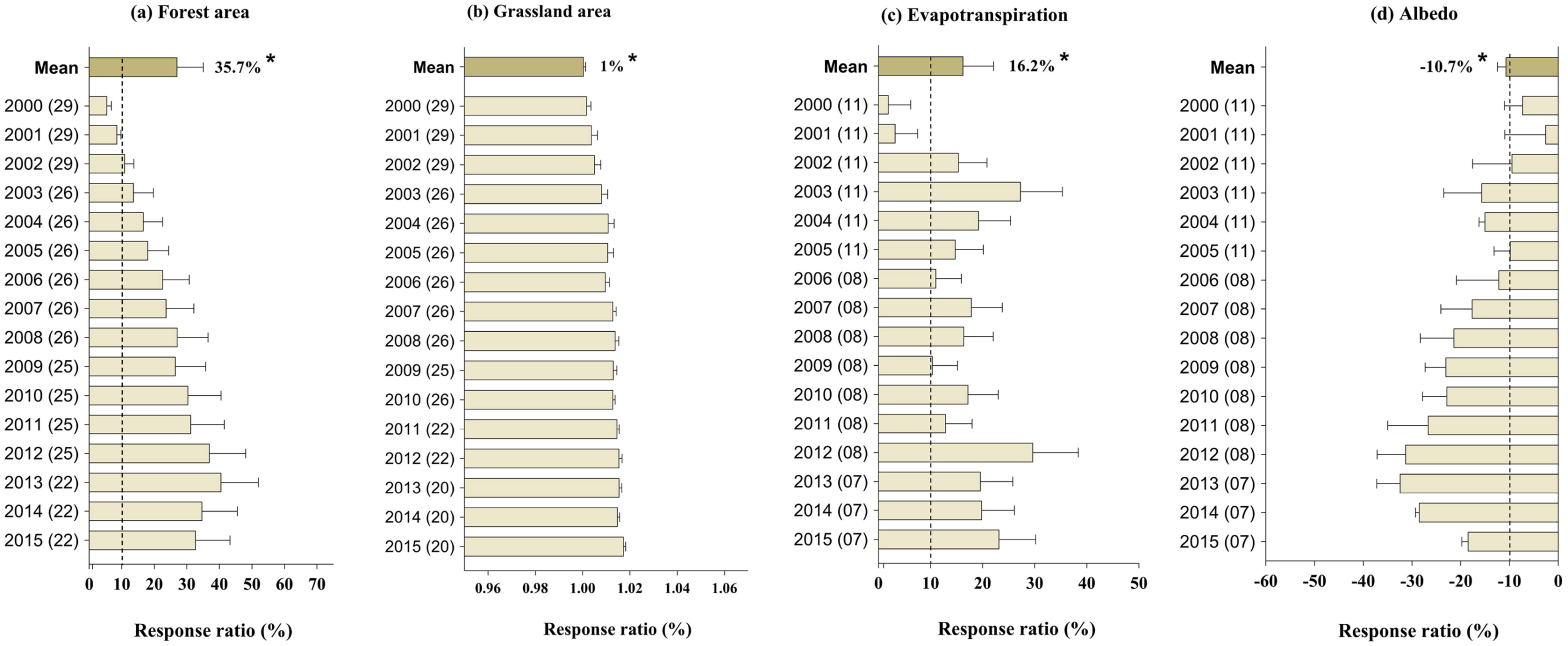
Response ratios of: (A) forest area, (B) Grassland area, (C) evapotranspiration, (D) and albedo to GFGP implementation between 2000 and 2015. Black bars represent 95% confidence intervals. The different letters in parentheses represent the number of observations. The dashed vertical line was drawn at a mean effect size of 10%. *Correlation is significant at the 0.05 level.

When averaged across studies, the GFGP has a positive and significant correlation with forest coverage by 35.7% (95% CI [24.15–47.52%]) (*p* < 0.01, Fig. 1A) from 2000 to 2015. The response ratio of grassland showed a significant increase by an average of 1.05% (95% CI [0.8–1.28%]) (*p* < 0.01, Fig. 1B). When averaged across studies, GFGP increased the rate of evapotranspiration response by 16.2% (95% CI [12.39–20.11%]) (Fig. 1C). In contrast, the effect of GFGP implementation was significant and negative, with albedo by an average of 10.7% (95% CI [9.55–11.88%]) (*p* < 0.01, Fig. 1D). Regarding their interaction, both forest coverage and grassland significantly increase (*p* < 0.01, Fig. 1). The response of evapotranspiration to GFGP increased with time (*r* = 0.77, *p* = 0.18, Fig. S5A), while it was negative and significant to albedo response (*r* = −0.89, *p* < 0.01, Fig. S5A).

### Effect of GFGP on soil conservation

**Figure 2:**
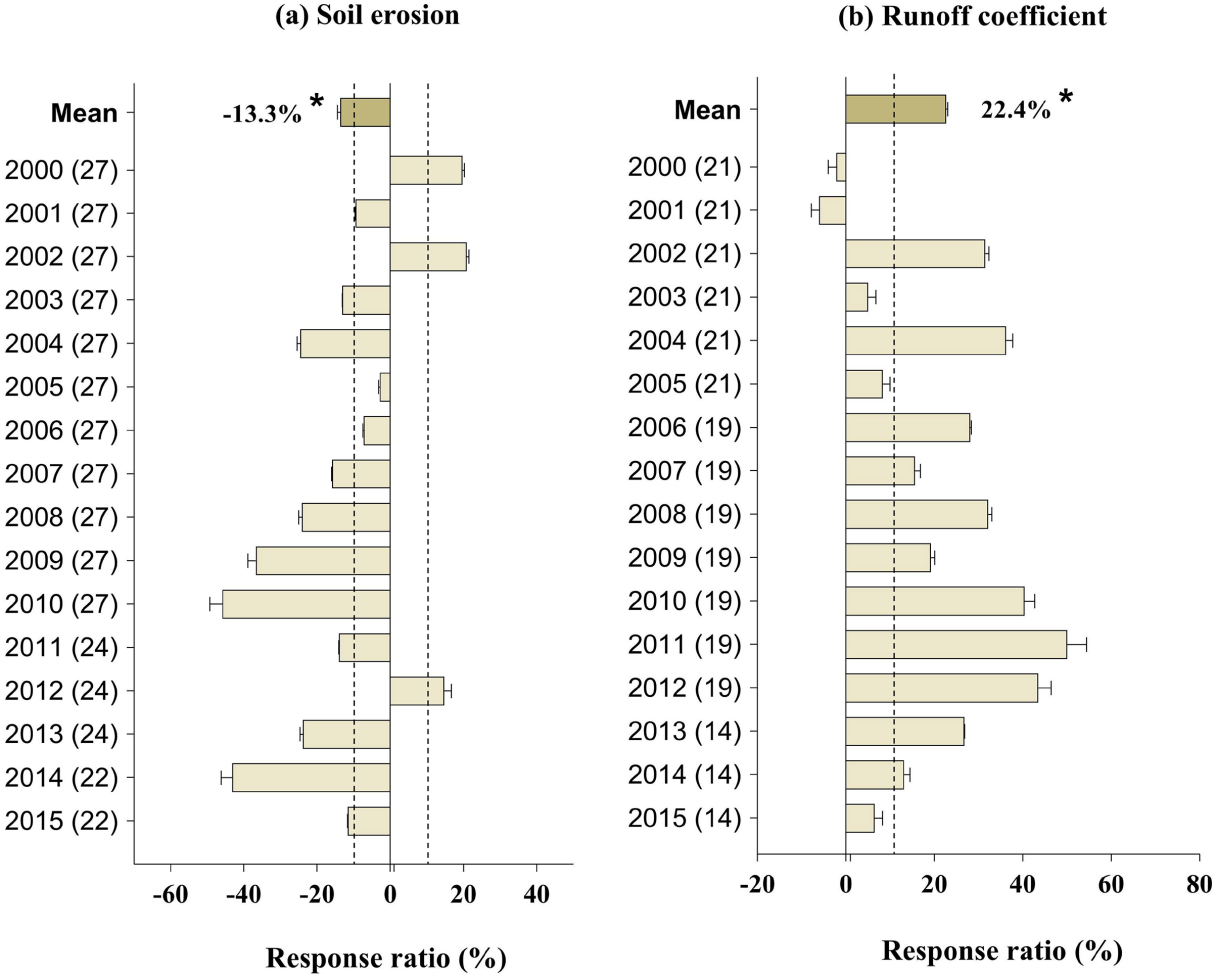
Response ratios of soil erosion (A) and runoff coefficient (B) to GFGP implementation between 2000 and 2015. Black bars represent 95% confidence intervals. The different letters in parentheses represent the number of observations. The dashed vertical line was drawn at a mean effect size of 10%. *Correlation is significant at the 0.05 level.

GFGP implementation reduced soil erosion by 13.3% (95% CI [0.27–25.76%]) (*p* < 0.05, Fig. 2A), while it slightly reduced runoff coefficient by 22.4% (95% CI [5.28–40.45%]) (Fig. 2B). When averaged across the restoration age, GPGP significantly reduced soil erosion (Fig. 2). The soil erosion and runoff coefficient responses were negatively correlated with the forest coverage response, but it was significant only for soil erosion (Fig. S5B; *r* = −0.50, *p* < 0.01 and *r* = −0.86, *p* = 0.88).

### Effect of GFGP on water resource

**Figure 3:**
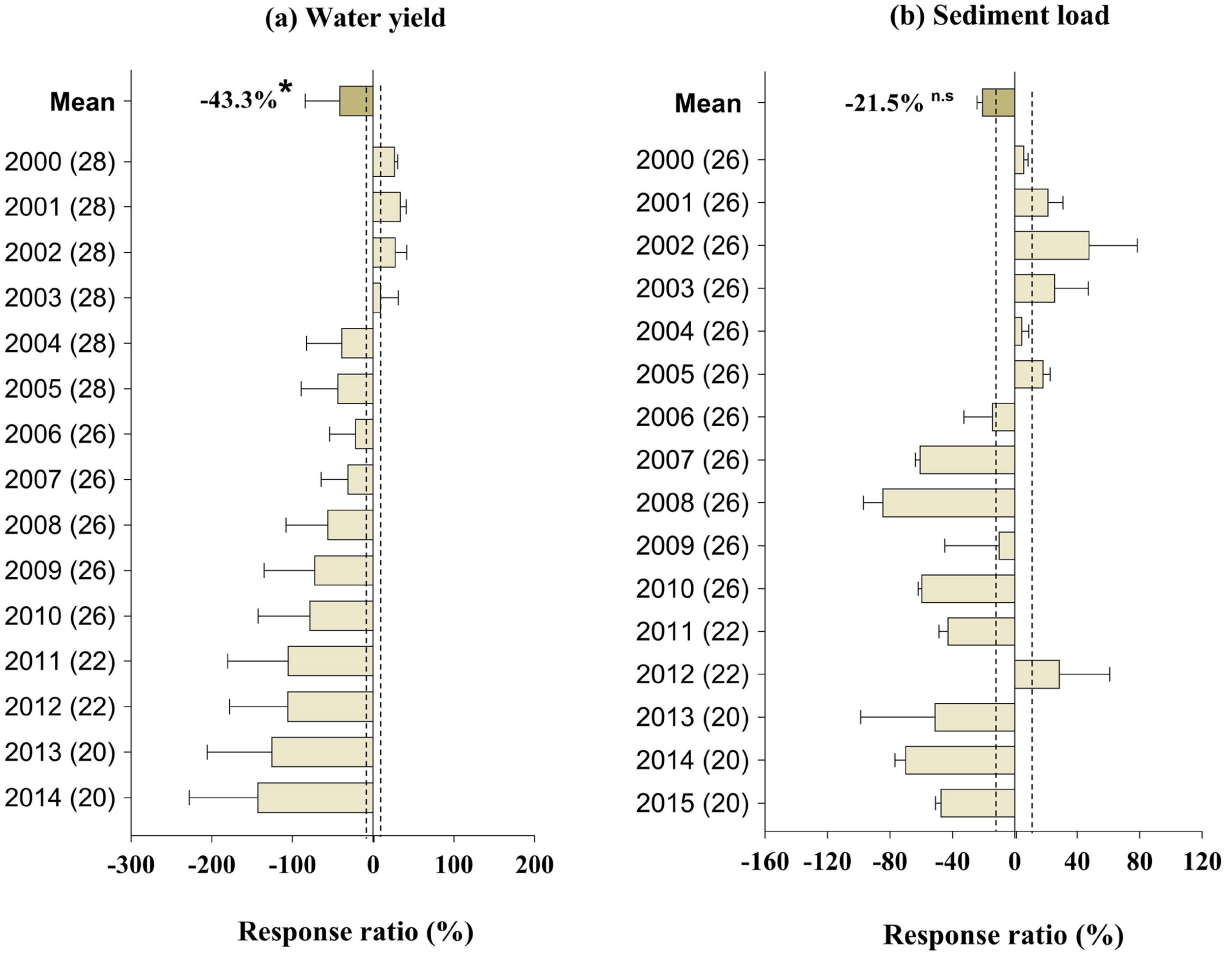
Response ratios of water yield (A) and sediment load (B) to GFGP implementation between 2000 and 2015. Black bars represent 95% confidence intervals. The different letters in parentheses represent the number of observations. The dashed vertical line was drawn at a mean effect size of 10%. *Correlation is significant at the 0.05 level.

Water yield was significantly decreased by an average of 43.3% (95% CI [27.03–82.86%]) (*p* < 0.01, Fig. 3A). In comparison, the decrease in sediment load rate by an average of 21.5% (95% CI [1.50–39.99%]) (Fig. 3B) showed a negative and significant correlation with restoration age (*p* < 0.01, Fig. 3). The increase in forest coverage following GFGP implementation showed a significant and negative correlation with sediment load and water yield (*r* = −0.73, *p* < 0.05 and *r* = −0.86, *p* < 0.01, respectively) (Fig. S5C).

### Effect of GFGP on carbon fluxes and sequestration

**Figure 4:**
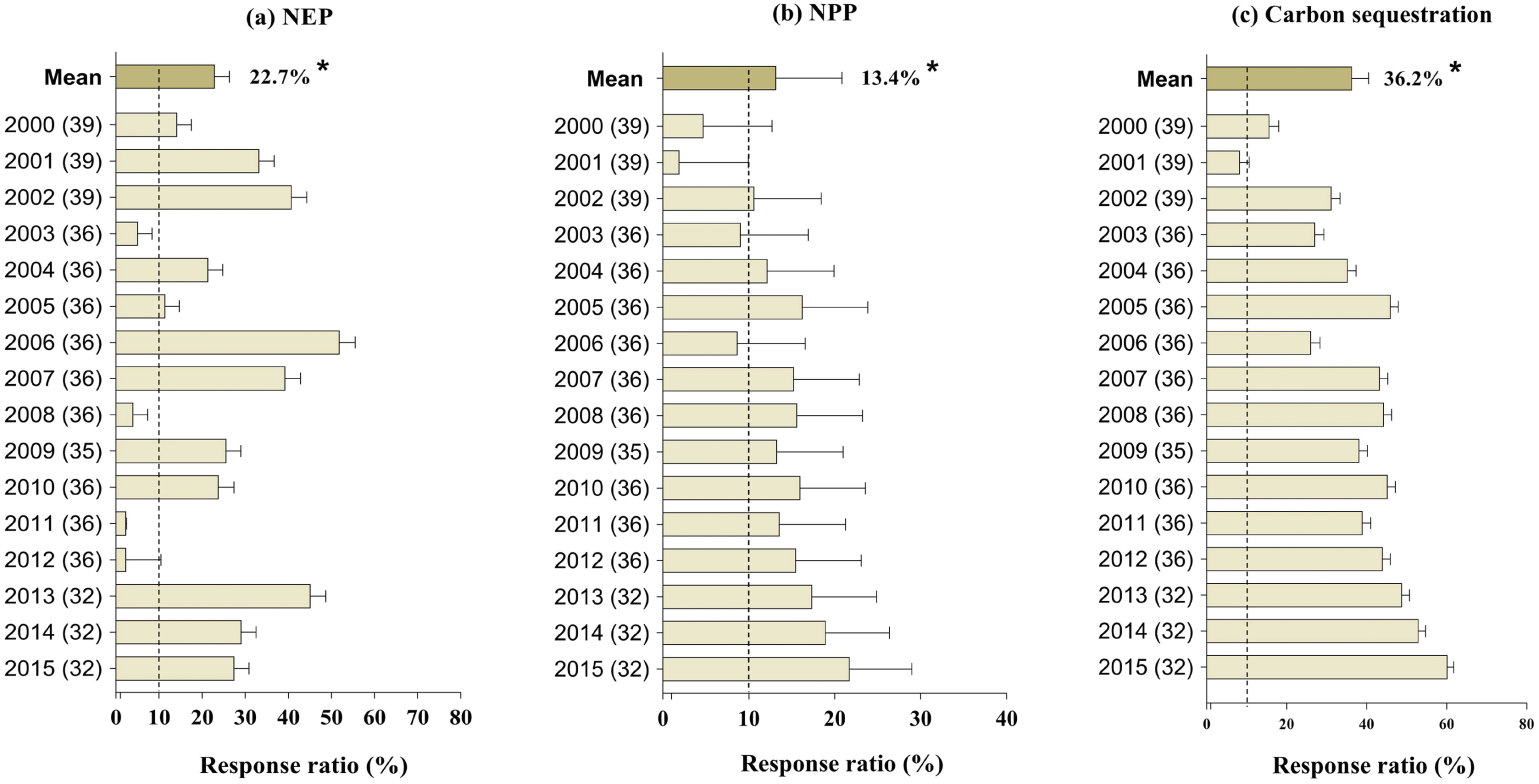
Response ratios of net ecosystem production (NEP) (A), net primary production (NPP) (B), and C sequestration (C) to GFGP implementation between 2000 and 2015. Black bars represent 95% confidence intervals. The different letters in parentheses represent the number of observations. The dashed vertical line was drawn at a mean effect size of 10%. *Correlation is significant at the 0.05 level.

The GFGP significantly increased NEP, NPP, and soil C sequestration, from 2000 to 2015 by an average of 22.7% (95% CI [9.10–36.79%]), 13.5% (95% CI [9.44–17.354%]), and 36% (95% CI [28.96–43.18%]), respectively (*p* < 0.05 for all, Figs. 4A–4C). The response ratio of NEP and NPP were positive and significantly correlated with restoration age (*r* = 0.75, *p* < 0.01; and *r* = 0.55, *p* < 0.01; Fig. 4). Similar observations have been made for soil C sequestration. As indicated in Fig. S5C, the increase in forest coverage following GFGP implementation was significant and positively correlated with soil C sequestration, NPP, and NEP (*r* = 0.76, *p* < 0.01; *r* = 0.57, *p* < 0.01; and *r* = 0.77, *p* < 0.01, respectively) (Fig. S5D).

### Effect of ecosystem services impact on water resources availability

**Figure 5:**
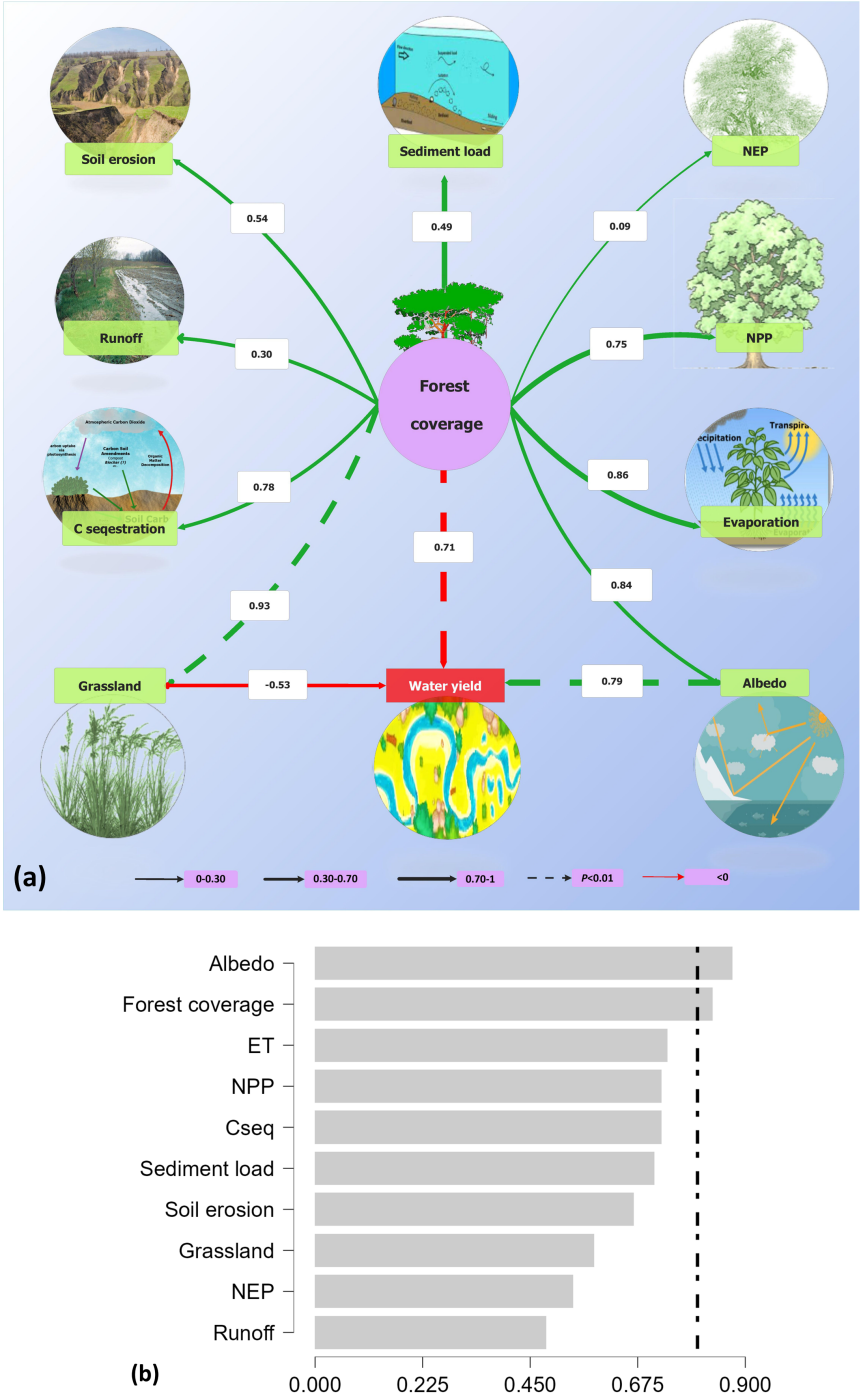
Structural equation model (SEM) evaluating the direct and indirect effects of forest coverage on other environmental outcomes under GFGP (A), and model-averaged importance of the predictors of the effect of the environmental outcomes (B) on wate. (A) Dashed lines indicate the relationship is significant at *p* < 0.01 level; lines indicate the relationship is not significant at *p* = 0.05 level; line thickness represents the magnitude of the strength of the relationship. Red lines indicate a negative correlation, while black lines a positive. Numbers adjacent to arrows are standardized path coefficients, indicating the effect size of the relationship. (B) The cutoff is set at 0.8 (dashed line) to differentiate between the important and unimportant predictors where the significance level was set at α = 0.05. Evapotranspiration (ET), net ecosystem production (NEP), net primary production (NPP), and C sequestration (Cseq). Correlation is significant at the 0.05 level.

Our study revealed that water resource availability is driven and dominantly affected by albedo, forest coverage, and grassland in the Loess Plateau, whereby forest coverage implementation increased but as water resources decreased (Fig. 5A). According to the structural equation model (SEM) suggests that increased ecological restoration indirectly accelerated water resource shortages *via* grassland. In addition, increasing forest area through tree plantation directly and positively affects soil erosion, sediment load, NEP, NPP, runoff, C sequestration, albedo, and evaporation rate. Our findings also suggested that water resource availability is driven by albedo and forest coverage in the Loess Plateau, whereby forest coverage implementation increased but as water resources decreased (Fig. 5B). Moreover, the model-averaged revealed that albedo and forest coverage increase were the main controlling factors of water resources. The previous relationships were consistent with our linear regression analysis as shown in Fig. S5, which also revealed that water resources were negatively correlated with increased forest coverage and grassland.

## Discussion

### Benefits of GFGP implementation on environmental outcomes

Forest ecosystems are the most effective land-use types in controlling erosion *via* the canopy protection of the soil’s surface from splash and surface sealing. Tree planting is a common approach to restoring degraded ecosystems (Zaady, Shachak & Moshe, 2001). Our meta-analysis showed that forest coverage and grassland response to Grain for Green Project (GFGP) implementation were positively correlated with time and increased by 37.1% and 1%, respectively. Similarly, previous studies found that the area of forest and grassland increased by 0.3–32.15% and 0.8–2.23%, respectively (Cao et al., 2019; Jiang, Zhang & Zhang, 2018; Shi et al., 2020). Accordingly to Wang et al. (2018), the GFGP almost doubled the forest area of the LP in 16 years (2001–2016), with a net forest gain of 49.248 km^2^. This is likely due to the high and large reforestation rate, with an average ratio of 80–81.2 million trees planted per year (Zhou & Van Rompaey, 2009) and the use of fast-growing tree species.

Vegetation can protect soil against erosive agents and fix the soil through mechanical, thermal, and hydrological regulation. Previous studies have pointed out that soil conservation has improved significantly since implementing GFGP through large-scale vegetation restoration (Liu et al., 2020; Wu et al., 2019b; Zhou & Van Rompaey, 2009). In line with our findings, Fu et al. (2011) and Wu et al. (2019a) demonstrated that soil erosion had a downward trend of 0.96 × 10^8^ t year^−1^ from the years 2000 to 2015 and a decrease of 34% from 2000 to 2008 (Fu et al., 2011; Wu et al., 2019a). We found that soil erosion significantly declined by 13.3% (*p* < 0.05, Fig. 2A), probably due to the development stage or understory vegetation growth status, and plays a crucial role in intercepting rainfall and retaining water (García-Ruiz, 2010; Oliveira, Wendland & Nearing, 2013; Wang et al., 2021) and *via* the canopy protection of the soil’s surface from splash and surface sealing. Vegetation cover insulates the soil surface from the impact of raindrops by blocking or intercepting precipitation, reducing erodibility, and stabilizing soil aggregates (Ngaba, Bol & Hu, 2021; Wei et al., 2009; Zhou et al., 2016).

On the other hand, as indicated by Fig. 3B, increasing vegetation coverage decreases sediment load by protecting the soil surface during rainy seasons by slowing water and sediment flows. It is well known that an unprotected surface can lead to more efficient sediment transport. The rainfall intensity and frequency will break up the soil structure on the surface and reduce the number of obstacles to surface flow. Also, the total amount of soil loss was estimated at 1.51 billion tons in 2008, compared to 2.07 billion tons in 2000 (Fu et al., 2010; Ma, Zhu & Zhao, 2020). The sediment load negatively and significantly decreased with time (*p* < 0.05, Fig. 3B). Wu et al. (2019a) found that sediment load between Tongguan and Toudaoguai stations decreased significantly by 0.25 × 10^8^ t year^−1^ from the years 2000 to 2015, while Sun, Shao & Liu (2013) showed that soil loss had reduced considerably to about 1–3 t hm^−2^ a^−1^ in the LP. Erosion rates are recorded mainly in ravine and hill regions like those in the southern part of Ningxia Province. Similarly, Wu et al. (2019c) reported that the soil conservation capacity showed a positive trend of 1.54 t ha^−1^ year^−1^. This result demonstrates that vegetation coverage reduces sediment detachment and nutrient transport. However, soil conservation capacity varies among different ecosystems.

Our meta-analysis showed that runoff was reduced by 22.4%, which is not far from the result reported by Huang, Zhang & Gallichand (2003), who indicated that runoff declined by 32% in the LP. Besides, the runoff coefficient in all LP watersheds has decreased in recent decades because of management practices such as terracing (Jiang, Zhang & Zhang, 2018; Wen & Zhen, 2020). Feng et al. (2020) found in the Ansai watershed that runoff was influenced by precipitation, vegetation, topography, soil properties, and supporting practices. This might explain why soil retention varies with precipitation rates on the LP (Fu et al., 2011). These outcomes, therefore, demonstrate that afforestation is an effective and appropriate way to increase aggregate stability and decrease soil erodibility (An, Darboux & Cheng, 2013; Fu et al., 2011; Ngaba, Bol & Hu, 2021). According to Nunes (2011), runoff coefficient and sediment load decline probably because there is no strong resistance to water infiltration into the soil matrix due to microporosity created by roots and soil biota. Feng et al. (2013) reported a decrease in runoff in the northeast to the southwest area of the LP with an average of 10.3 mm year^−1^ over the entire Loess Plateau between 2002 and 2008. Such results suggest that [high planting density is the primary cause of severe soil moisture deficit (An et al., 2017) and forest vegetation has high water demand (Fang et al., 2017; Fu et al., 2011; Lü et al., 2012). Implementing a sustainable restoration project, especially in semi-arid regions, requires an approach that considers the vegetation to be established and planting density based on local soil moisture conditions.

Soil C sequestration is an essential and efficient index for assessing the effectiveness of ecological restoration. In China, reforestation through ecological restoration represents a significant positive contributor to soil carbon (C) storage and carbon sequestration from vegetation in recent years (Fu et al., 2010; Ge et al., 2020), consistent with our results. We found that GFGP significantly increased soil C sequestration by 36% from 2000 to 2015. According to Wu et al. (2019a) and Feng et al. (2013), over 90% of the Loess plateau (LP) increased C sequestration with an average annual growth ranging between 8.5 g C m^−2^ year^−1^ and 11.50 g C m^−2^ year^−1^ from 2000 to 2015. However, there has been an ongoing debate on whether the land use type within a forest, grassland, or shrubland promotes a greater accumulation rate of soil C. Tuo et al. (2018) reported that grassland restoration resulted in greater soil organic C (SOC) and total nitrogen accumulation than shrub and forest areas where mean annual precipitation was less than 510 mm. Other studies shed more light on this issue. By assessing the restoration programs impact on SOC, Chen et al. (2007) reported that shrubland was more effective than grassland in SOC accumulation, while Wang et al. (2016) indicated that SOC enrichment was higher in forestland than in shrubland with 39.8% and 10.8%, respectively. These discrepancies observed between these results are probably since soil C accumulation is influenced by biotic and abiotic factors processes such as soil types, management practices, vegetation type, soil depth and respiration, soil physicochemical properties, litter or biomass supply, among others, hence different rates of C release to the soil (Deng et al., 2016; Deng et al., 2018; Li et al., 2016; Ngaba, Ma & Hu, 2020; Wang et al., 2016; Zhang et al., 2013). Linked to this, evidence suggests it is essential to plant new forests and simultaneously maintain the existing forests because a well-maintained forest absorbs more carbon than an unmaintained one.

### Factors influencing the effectiveness of GFGP and water resources drivers

Although vegetation cover restoration promoted vegetation growth, C sequestration, and soil erosion control, this measure also negatively impacted other ESs, such as the water cycle (Fig. 5). Our study indicated that GFGP significantly decreases water yield and evapotranspiration by 43.3% and 16.2%, respectively (Fig. 3A). Evapotranspiration is a major green (part of water available for plant use) water flux. Its increase probably results from tree growth, which consumes more water than shorter plants, reducing the amount of water flowing into rivers and lakes or recharging groundwater. Besides, forest ecosystems have higher water consumption (*via* root water uptake) than grassland and shrubland (Feng et al., 2020). Our results agree with earlier studies done by Lü et al. (2012) and Jiang, Zhang & Zhang (2018), which found that afforestation compromised other ES, such as hydrological regulation. Wang et al. (2017) revealed a soil moisture deficit after afforestation, while Feng et al. (2020) indicated a reduction in water yield by 37.6% from 2000 to 2014. Several mechanisms, abiotic and biotic factors such as infiltration, tree species, and vegetation restoration selected can be attributed to the decrease (Fig. 5). In general, water infiltration, vegetation structure, and soil quality improvement occur during the restoration process, reducing soil loss and moisture (Jiang, Zhang & Zhang, 2018; Zhou et al., 2016). Besides, the fast and large-scale GFGP implementation is a factor because it led to increased water consumption and decreased water accessibility and availability for both human and natural ecosystems (Geng et al., 2020; Jiang, Zhang & Zhang, 2018). In congruence with this explanation, this study reported a strong correlation between evapotranspiration and forest coverage (*p* < 0.05, Fig. 1C). The solution should be to gradually replace high water-consuming exotic species with indigenous species (choose native) to avoid climate adaptation or use natural vegetation recovery as a management option (Jiao et al., 2012; Wei et al., 2017; Wu et al., 2019a).

Moreover, GFGP-induced forest plantations are generally composed of a few improved, fast-growing, exotic, and uniformly planted species of the same age. This approach has serious drawbacks and may increase ecological risks such as invasiveness, hybridization, and the appearance of pathogens and pests. Indeed, these monoculture plantations are less resilient to climate change because fires, excessive droughts, and heat could make them more vulnerable. Moreover, these forests are not compatible with the ecological and social needs of the people living in and around these areas. Besides, exotic species have been preferred compared to local species. It remains a challenge to provide guidelines for the appropriate use of exotic species in forest management and land restoration that consider the risks associated with species biodiversity. Many projects have highlighted the problems associated with the use of these species. The introduction of an exotic species poses genetic risks to neighboring native species, including interspecific competition and genetic contamination (Barabás, Michalska-Smith & Allesina, 2016). Though this phenomenon is part of species’ evolution and helps create better-adapted individuals, the crossbreeding between exotic and native species could produce even more vigorous individuals that, in turn, are capable of invading the environment (Ayres et al., 1999; Hoddle, 2004). The invasion of exotic species into an ecosystem is related to their ability to grow and expand rapidly in sites where local species perform less well because they are less sensitive to environmental conditions (Thomas & Reid, 2007). The disturbance of habitat is often the precursor for a species to invade it, which makes using these species more of a threat to any open or disturbed peripheral habitats or disturbed habitats. According to Dodet & Collet (2012), the type and degree of disturbance determine the established species and invasion dynamics. Therefore, it is recommended to promote the restoration of natural forests with biological diversity or use local species. Such an approach can help restore and feed biodiversity, enhance and better secure carbon storage, especially in the soil, because forests with biological diversity are less vulnerable than monocultures to fire pests and drought (Achat et al., 2015; Felton et al., 2016).

In congruence with this explanation, this study highlighted that an increase in vegetation cover is accompanied by an increase of evapotranspiration (*p* < 0.05, Fig. 1C), a decrease in albedo (*p* < 0.05, Fig. 1D) and water yield (*p* < 0.05, Fig. 4A). In addition, water yield pattern is predicting by albedo and forest coverage in the loess plateau (Fig. 5B). These changes can be explained by modifying land surface biophysical properties such as vegetation fractions, leaf area index, surface albedo, soil rugosity, and soil moisture (Guo, Xie & Subrahmanyam, 2019). Further research has shown that the mechanisms that demonstrate the link between water yield and albedo. According to Garratt (1993), albedo directly impacts land evaporation, land precipitation, and precipitation over the sea in the global change cases. Charney, Stone & Quirk (1975) showed that the precipitation rate in Sahara under the low-albedo experiment (0.14) was higher than under the high-albedo experiment (0.35) by 4.4 mm day^−1^ and 2.5 mm day^−1^ respectively. In the same line, Cao et al. (2019) point out that the vegetation restoration has strongly influenced climate by increasing summer precipitation in the Southeastern LP area by 1.0–2.0 mm day^−1^ and decreasing summer precipitation in Western LP and Northern Shanxi Province by 1.0–1.4 mm day^−1^. At the same time, a decrease in precipitation rate leads to an increase in aridity and, therefore, to a decrease in soil, land, and air moisture. In addition, there are evidence links of surface albedo changes to soil moisture, evapotranspiration, and annual precipitation because arid regions are usually characterized by low precipitation and high-incoming solar radiation. Hence, a minor or significant alteration of surface vegetation might affect all ecosystems (Deng et al., 2016). Variations in albedo in the LP are caused by shifts in herbaceous cover, whose production is influenced by the allocation of water resources during the rainy season. Therefore our findings support the statement that albedo is also a key driver of local climate, which, in turn, will affect water resources.

### Limitations of study and uncertainties of data sources

Although our meta-analysis makes important contributions to the literature regarding the effect of the revegetation due to GFGP on soil erosion, C sequestration, and water yield, it has some limitations. Firstly, other forest restoration programs were already implemented in the LP before the GFGP. Despite the results observed after their implementation, it is difficult to attribute all their impacts to the GFGP project alone. Secondly, the choice of databases through the selection criteria may have affected the number of relevant articles. Indeed, other studies have been carried out on the LP, but a more holistic database would have reinforced the findings. This may also lead to uncertainties regarding the representativeness of the themes. The environmental conditions in different regions vary considerably. Furthermore, the documentary research was based solely on electronic data. Therefore, governmental or institutional reports, academic theses, and books were omitted, which may impact the integrity of this review.

## Conclusion

This meta-analysis helps to identify the impact of the measures implemented for the project and future research work. The LP area has seen improvements, including improved soil conservation and the expansion of grasslands and woodlands, whereas water fluxes have changed over time in the Loess Plateau. Overall, our study indicated that changes in forest coverage lead to changes in surface albedo and in turn, negatively affect water resources. In addition, the water shortage can be attributed to afforestation and could also be induced by the warmer and wetter climate variations on the LP. A critical driving process of these results are the decline of runoff and the increase of evapotranspiration. This imbalance in trade-off responses between the multiple ESs represents a threat to the LP ecosystem’s efficient and sustainable restoration because water resources are under threat from project management, which has a detrimental effect on good practices. Thus, it is crucial to maintain a stable relationship between water supply and other ESs to create win-wins from services in the Loess Plateau. It shows that GFGP’s land-use strategy needs to be adjusted because its impact on water supply is becoming an increasingly urgent issue for water resource managers and planners.

## Supplemental Information

10.7717/peerj.13658/supp-1 Supplemental Information 1 Raw data.

10.7717/peerj.13658/supp-2 Supplemental Information 2 Graphical abstract.

10.7717/peerj.13658/supp-3 Supplemental Information 3 Location of the Loess Plateau, China.

10.7717/peerj.13658/supp-4 Supplemental Information 4 Year-wise classification of publications.

10.7717/peerj.13658/supp-5 Supplemental Information 5 Showing the framework of the review methodology.

10.7717/peerj.13658/supp-6 Supplemental Information 6 Number of selected articles published in the LP.

10.7717/peerj.13658/supp-7 Supplemental Information 7 Response ratio of forest coverage on (A) evapotranspiration and albedo; (B) soil erosion and runoff coefficient; C flux: (C) sediment load and water yield, and (D) NEE and NPP and sequestration to forest restoration between 2000 and 2015. Lines represent. Lines represent mean, and the color band represents 95%, confidence band.

10.7717/peerj.13658/supp-8 Supplemental Information 8 Changes of variables resulting from GFGP in the Lœss Plateau.

10.7717/peerj.13658/supp-9 Supplemental Information 9 Ecosystem services assessment, data types and description of the selected articles.

## Additional Information and Declarations

### Data Availability

The following information was supplied regarding data availability: The raw data is available in the Supplemental File.
