# Correction: Ecological restoration stimulates environmental outcomes but exacerbates water shortage in the Loess Plateau

**DOI:** 10.7717/peerj.13658/correction-1

**Published:** 2022-09-15

**Authors:** Mbezele Junior Yannick Ngaba, Yves Uwiragiye, Hongzhi Miao, Zhiqin Li, Paulo Pereira, Jianbin Zhou

**Affiliations:** 1 College of Natural Resources and Environment, Northwest A&F University, Yangling, Shaanxi, China; 2 Ministry of Agriculture, Key Laboratory of Plant Nutrition and the Agri-Environment in Northwest China, Yangling, Shaanxi, China; 3 Crop production, University of Technology and Arts of Byumba, Byumba, Northern, Rwanda, Rwanda; 4 Environmental Management Laboratory, Mykolas Romeris University, Vilnius, Lithuania

Corrigendum:

Dr. Paulo Pereira was listed as a co-author in the original submission but did not respond to the journal’s attempts to confirm his co-authorship. He was removed prior to acceptance accidently as his coauthors believed he was unreachable to confirm his coauthorship.

After publication, Dr. Pereira contacted the authors to clarify that previous journal correspondence was mistakenly routed to his email spam folder.

As Dr. Pereira’s contributions qualify him for authorship, this has been corrected so that the author list is now: Mbezele Junior Yannick Ngaba, Yves Uwiragiye, Hongzhi Miao, Zhiqin Li, Paulo Pereira, Jianbin Zhou

The citation for this article has been updated to:

Ngaba MJY, Uwiragiye Y, Miao H, Li Z, Pereira P, Zhou J. 2022. Ecological restoration stimulates environmental outcomes but exacerbates water shortage in the Loess Plateau. PeerJ 10:e13658 https://doi.org/10.7717/peerj.13658

Original PDF

