# Supplementary figures and images for: Ecological restoration stimulates environmental outcomes but exacerbates water shortage in the Loess Plateau
Source: PeerJ. 2022 Jul 8;10:e13658. doi: 10.7717/peerj.13658 (PMC9272815; doi:10.7717/peerj.13658)

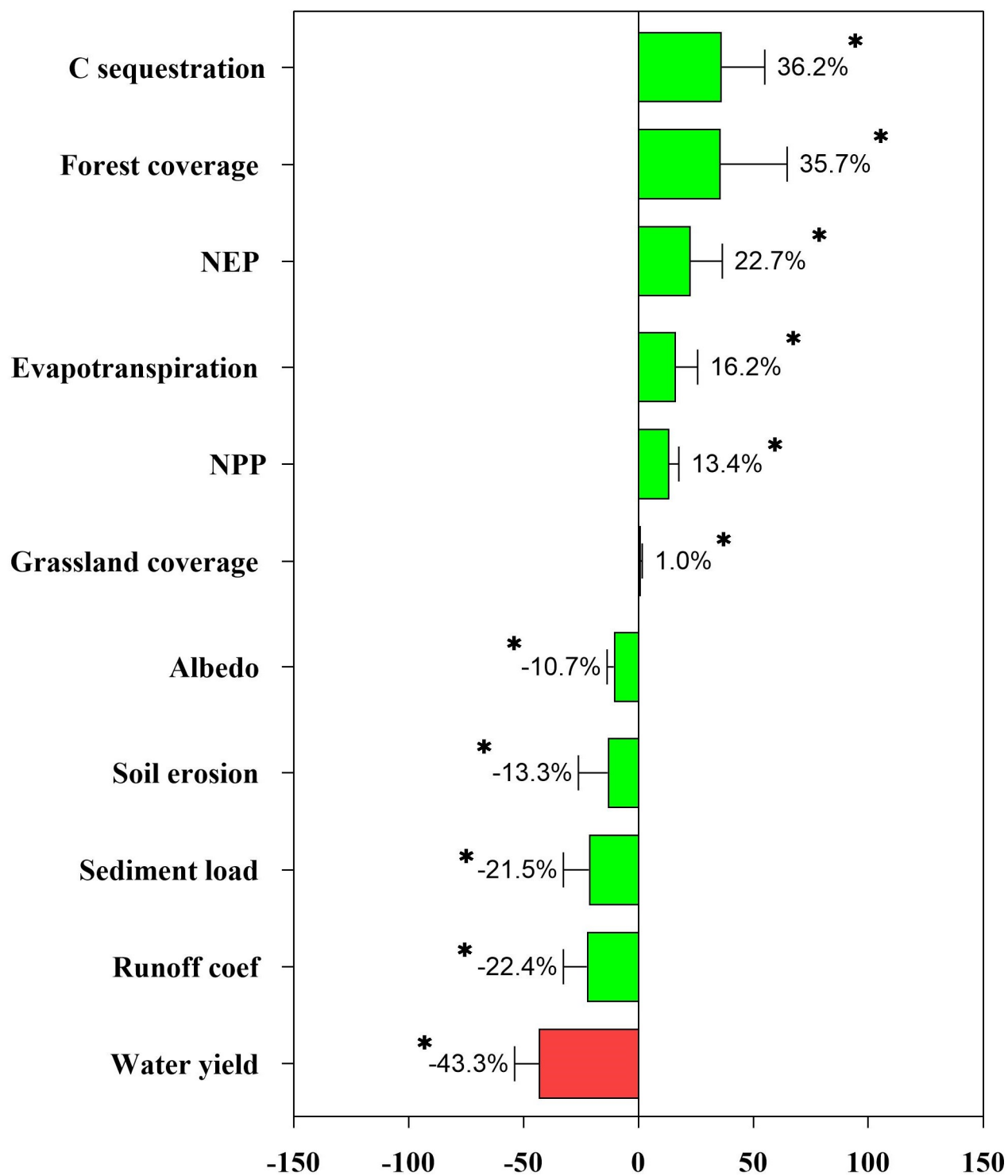

Supplement: Supplemental Information 2 [file peerj-10-13658-s002.pdf]

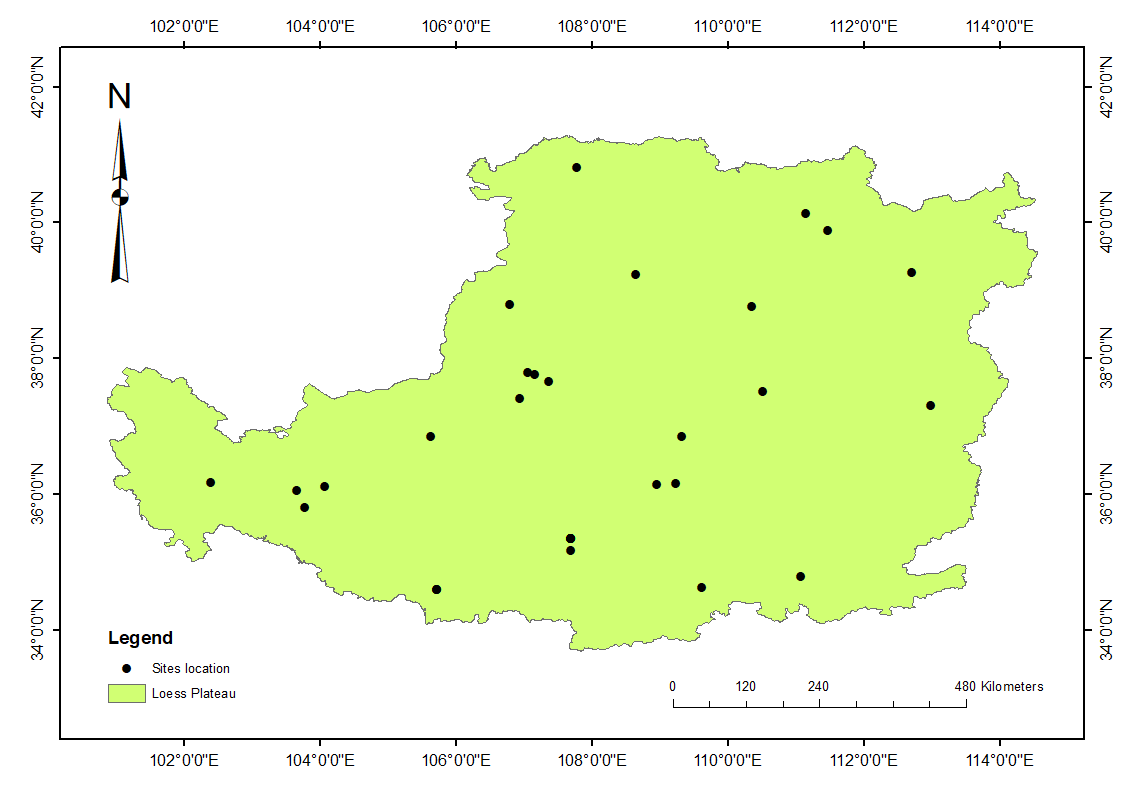

Supplement: Supplemental Information 3 [file peerj-10-13658-s003.png]

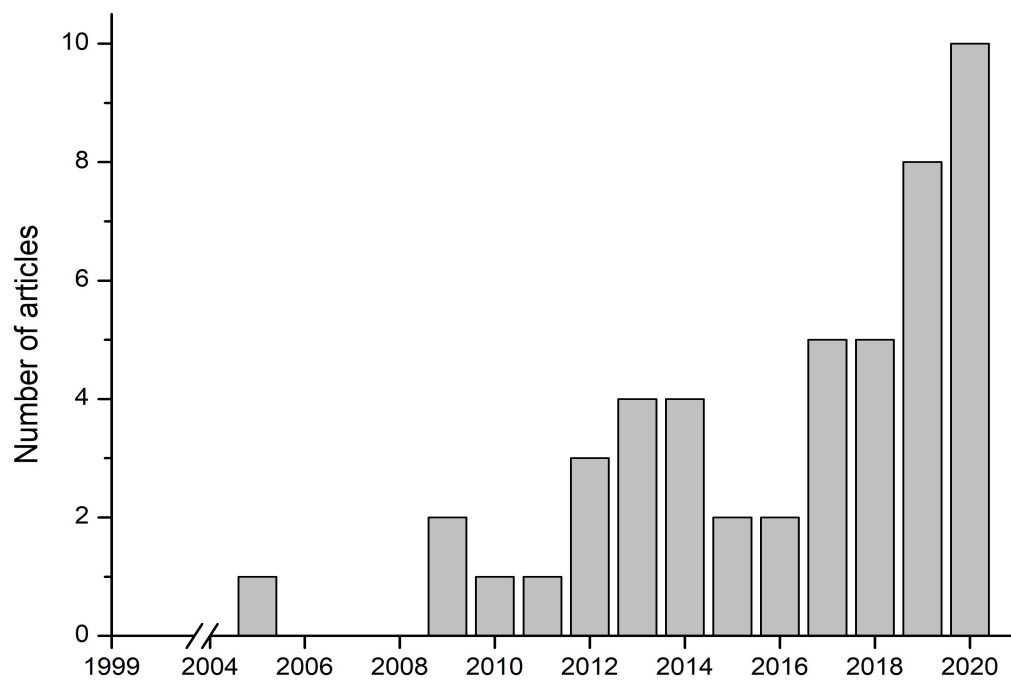

5  
6  
7

Supplement: Supplemental Information 4 [file peerj-10-13658-s004.pdf]

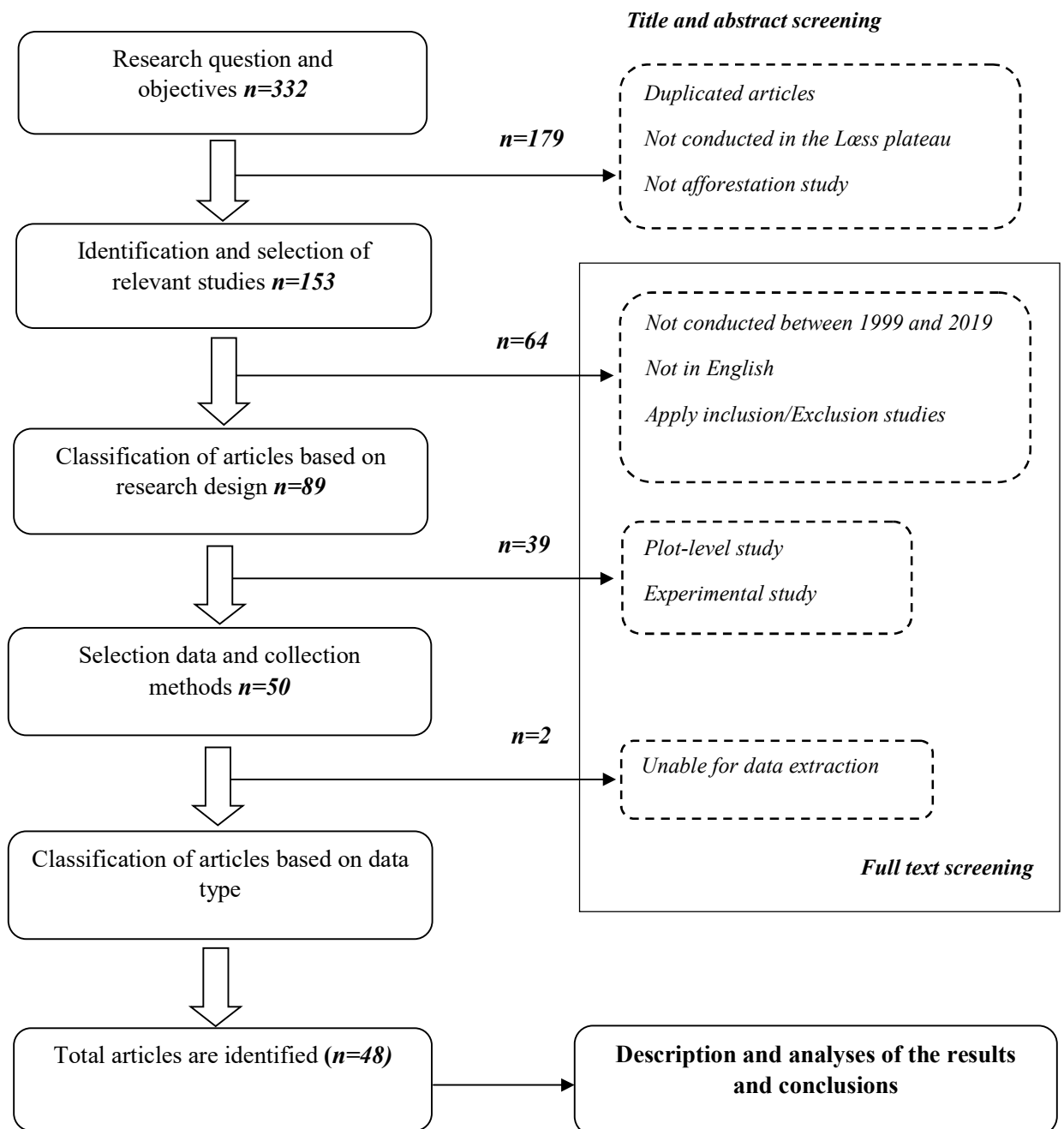

Supplement: Supplemental Information 5 [file peerj-10-13658-s005.pdf]

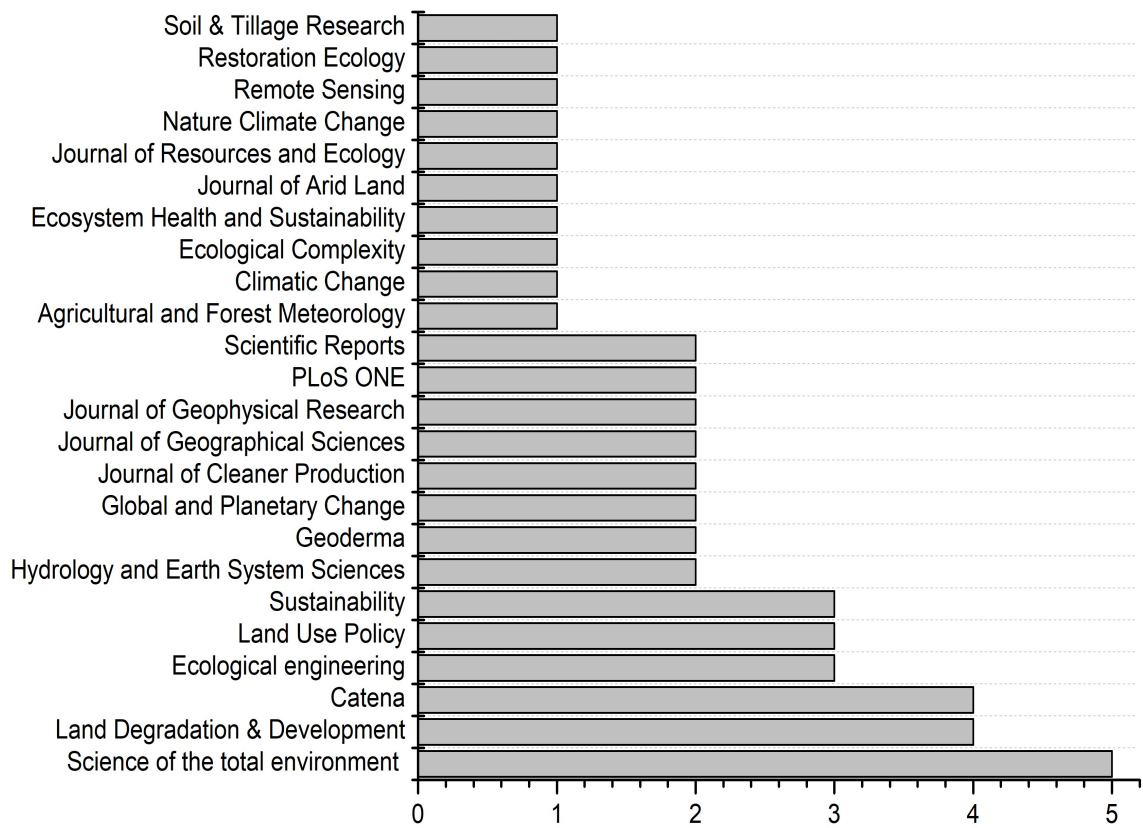

35

36

37

38

39

Supplement: Supplemental Information 6 [file peerj-10-13658-s006.pdf]

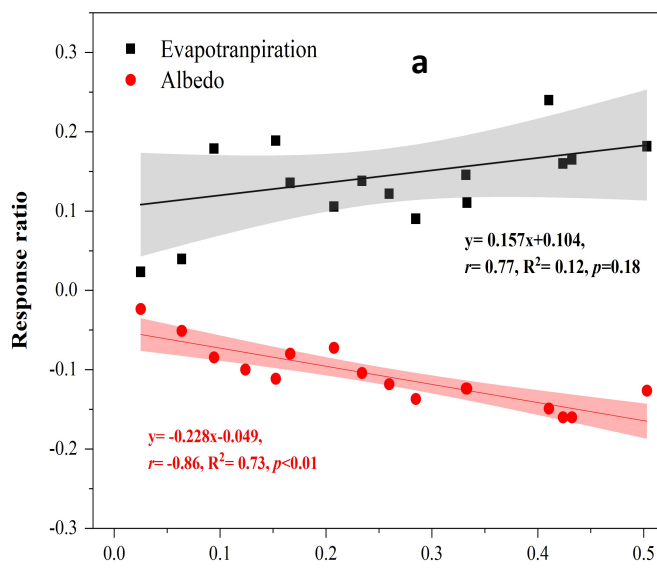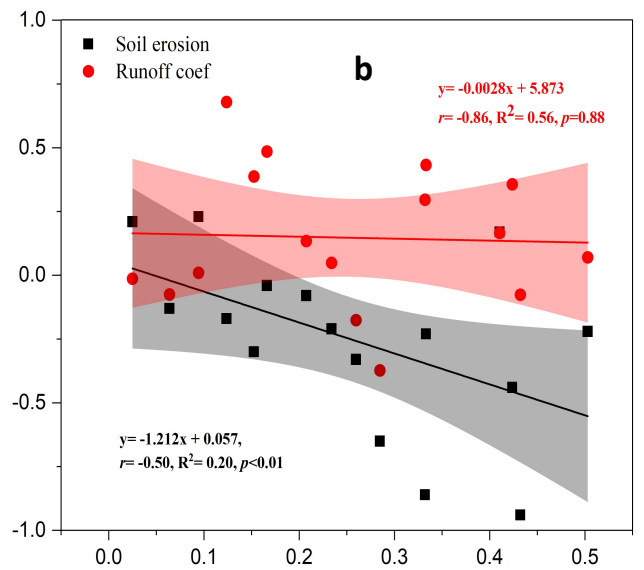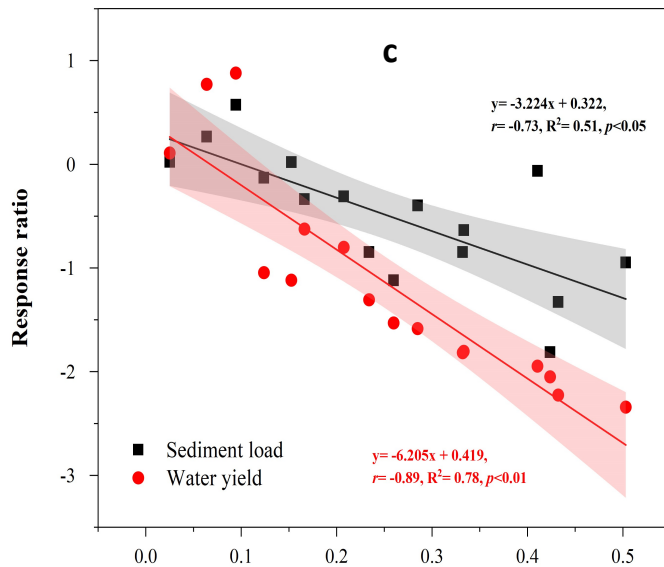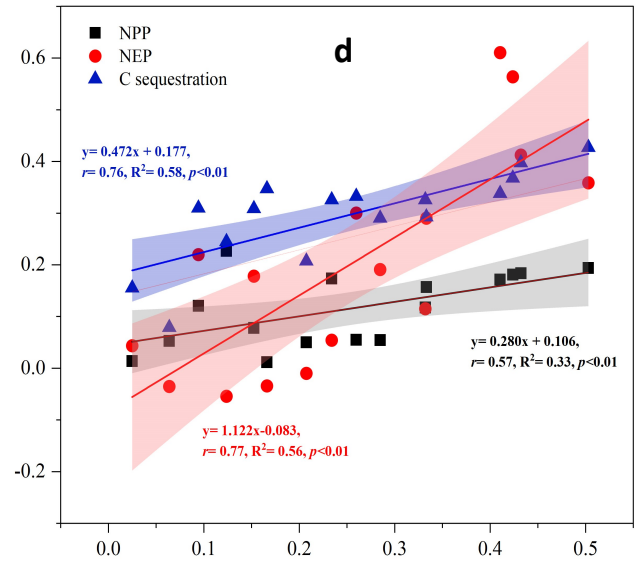

Response ratio of forest

Response ratio of forest

Supplement: Supplemental Information 7 — Lines represent mean, and the color band represents 95%, confidence band. [file peerj-10-13658-s007.pdf]
