# Supplementary material for: Ecological restoration stimulates environmental outcomes but exacerbates water shortage in the Loess Plateau
Source: PeerJ. 2022 Jul 8;10:e13658. doi: 10.7717/peerj.13658 (PMC9272815; doi:10.7717/peerj.13658)
Supplement: Supplemental Information 8 [file peerj-10-13658-s008.doc]

# Table S1. Changes of variables resulting from GFGP in the Lœss Plateau.

| **N°** | **Sites (County)** | **Province** | **Longitude** | **Latitude** | **Time scale** | **References** |
| --- | --- | --- | --- | --- | --- | --- |
| 1 | Anshai, | Shaanxi | 109° 19 ′E | 36° 51 ′N | 2016 | (Shi et al., 2020) |
| 2 | Baota District, Yan'an | Shaanxi | 109°14′-110° 50 ′E | 36°10′-37° 2 ′N | 2000, 2005  and 2010 | (Guo et al., 2015) |
| 3 | Jingbian, Ansai, Baota, Yanchang, Luochuan, Jingbian, | Shaanxi | 107°41′-110° 31 ′E | 35°21′-37° 31 ′N | 2005 | (Cao et al., 2009) |
| 4 | Liudaogou, Shenmu | Shaanxi | 110°21′-110° 23 ′E | 38°46′-38° 51 ′N | 2007 | (Fu et al., 2010) |
| 5 | Loess Plateau | Gansu, Henan, Shanxi, Mongolia, Ningxia, Shaanxi, Qianghai | 100°54′-114° 33 ′E | 33°43′-41° 16 ′N | 2005, 2010  and 2014 | (Geng et al., 2020) |
| 6 | Loess Plateau | Gansu, Henan, Shanxi, Mongolia, Ningxia, Shaanxi, Qianghai | 100°54′-114°33′E | 33°43′-41°16′N | 2000, 2015 | (Wu et al., 2019) |
| 7 | Loess Plateau | Gansu, Henan, Shanxi, Mongolia, Ningxia, Shaanxi, Qianghai | 100°54′-114°33′E | 33°43′-41° 16 ′N | 2000–2008 | (Lü et al., 2012) |
| 8 | Loess Plateau | Gansu, Henan, Shanxi, Mongolia, Ningxia, Shaanxi, Qianghai | 107°41′-110° 31 ′E | 35°21′-37° 31 ′N | 2000–2015 | (Wu, X. et al., 2019) |
| 9 | Loess Plateau | Gansu, Henan, Shanxi, Mongolia, Ningxia, Shaanxi, Qianghai | 103°-114 °E | 34°-40°N | 2000-2014 | (Wang et al., 2017) |
| 10 | Loess Plateau | Gansu, Henan, Shanxi, Mongolia, Ningxia, Shaanxi, Qianghai | 107°41′-110° 31 ′E | 35°21′-37° 31 ′N | 2000–2012 | (Xiao, 2014) |
| 11 | Loess Plateau | Gansu, Henan, Shanxi, Mongolia, Ningxia, Shaanxi, Qianghai | 107°41′-110°31′E | 35°21′-37°31′N | 2000-2004 | (Cao et al., 2019) |
| 12 | Loess Plateau | Gansu, Henan, Shanxi, Mongolia, Ningxia, Shaanxi, Qianghai | 107°41′-110° 31 ′E | 35°21′-39°34′N | 2000 and 2015 | (Deng et al., 2019) |
| 13 | Loess Plateau | Gansu, Henan, Shanxi, Mongolia, Ningxia, Shaanxi, Qianghai | 102-114°E | 32-41°N | 2001-2009 | (Fan et al., 2014) |
| 14 | Loess Plateau | Gansu, Henan, Shanxi, Mongolia, Ningxia, Shaanxi, Qianghai | 104°54′-114°33′E | 33°43′-41°16′N | 2000-2014 | (Zhao et al., 2019) |
| 15 | Loess Plateau | Gansu, Henan, Shanxi, Mongolia, Ningxia, Shaanxi, Qianghai | 100°54′-114°33′E | 33°43′-41°16′N | 2000 and 2010 | (Sun et al., 2013) |
| 16 | Loess Plateau | Gansu, Henan, Shanxi, Mongolia, Ningxia, Shaanxi, Qianghai | 100°52′- 114°33′E | 33°41′- 41°16′N | 2003-2013 | (Wang et al., 2018) |
| 17 | Loess Plateau | Gansu, Henan, Shanxi, Mongolia, Ningxia, Shaanxi, Qianghai | 100°54′-114°33′E | 33°43′-41°16′N | 2000 and 2008 | (Fu et al., 2011) |
| 18 | Loess Plateau | Gansu, Henan, Shanxi, Mongolia, Ningxia, Shaanxi, Qianghai | 100°54′-114°33′E | 33°43′-41°16′N | 2001-2017 | (Ge et al., 2020) |
| 19 | Loess Plateau | Gansu, Henan, Shanxi, Mongolia, Ningxia, Shaanxi, Qianghai | 107°41′-110° 31 ′ E | 35°21′-39° 34 ′ N | 2000–2008 | (Feng et al., 2013) |
| 20 | Loess Plateau | Gansu, Henan, Shanxi, Mongolia, Ningxia, Shaanxi, Qianghai | 107°41′-110° 31 ′E | 35°21′-39° 34 ′ N | 2000–2010 | (Feng et al., 2016) |
| 21 | Loess Plateau | Gansu, Henan, Shanxi, Mongolia, Ningxia, Shaanxi, Qianghai | 100°54′-114°33′E | 33°43′-41°16′N | 2000-2015 | (Gang et al., 2018) |
| 22 | Loess Plateau | Gansu, Henan, Shanxi, Mongolia, Ningxia, Shaanxi, Qianghai | 100°54′-114°33′E | 33°43′-41°16′N | 2000-2007 | (Feng et al., 2012) |
| 23 | Loess Plateau | Gansu, Henan, Shanxi, Mongolia, Ningxia, Shaanxi, Qianghai | 105° 43 ′E | 34°36′N | 2017 | (Liu et al., 2017) |
| 24 | Loess Plateau | Gansu, Henan, Shanxi, Mongolia, Ningxia, Shaanxi, Qianghai | 100°54′-114°33′E | 33°43′-41°16′N | 2000-2017 | (Su and Shangguan, 2019) |
| 25 | Loess Plateau | Gansu, Henan, Shanxi, Mongolia, Ningxia, Shaanxi, Qianghai | 100°54′-114° 33 ′E | 33°41′-41°16′N | 1999-2014 | (Lyu and Xu, 2020) |
| 26 | Loess Plateau | Gansu, Henan, Shanxi, Mongolia, Ningxia, Shaanxi, Qianghai | 105°43′E | 34°36′N | 2020 | (Wen and Deng, 2020) |
| 27 | Loess Plateau | Gansu, Henan, Shanxi, Mongolia, Ningxia, Shaanxi, Qianghai | 105°43′E | 34°36′N | 1999-2012 | (Deng et al., 2014) |
| 28 | Loess Plateau | Gansu, Henan, Shanxi, Mongolia, Ningxia, Shaanxi, Qianghai | 107°41′-110° 31 ′E | 35°21′-37° 31 ′N | 1999–2001 | (Shi et al., 2020) |
| 29 | Loess Plateau  (Yanchi, Dingbian, Wuqi, Zhidan, Yan'an, Jixian and Linfen (Loess Plateau) | Gansu, Henan, Shanxi, Mongolia, Ningxia, Shaanxi, Qianghai | 100°90′–114°55′E | 33°72′-41°27′N | 2016 | (Tuo et al., 2018) |
| 30 | Loess Plateau  Yellow River (Loess plateau) | Gansu, Henan, Shanxi, Mongolia, Ningxia, Shaanxi, Qianghai | 100°54′-114° 33 ′E | 33°43′-41° 16 ′N | 2000–2015 | (Fang et al., 2017) |
| 39 | Loess Plateau | Gansu, Henan, Shanxi, Mongolia, Ningxia, Shaanxi, Qianghai | 100°54′-114° 33 ′E | 33°43′-41° 16 ′N | 2000–2012 | (Jiang et al., 2018) |
| 40 | Loess Plateau | / | 96°21′-120° 23 ′E | 30°46′–42° 51 ′N | 2002–2016 | (Lv et al., 2019) |

#

# References

An, W., Li, Z., Wang, S., Wu, X., Lu, Y., Liu, G., Fu, B., 2017. Exploring the effects of the “Grain for Green” program on the differences in soil water in the semi-arid Loess Plateau of China. Ecol. Eng. 107, 144–151. https://doi.org/10.1016/j.ecoleng.2017.07.017.

An, W., Li, Z., Wang, S., Wu, X., Lu, Y., Liu, G., Fu, B., 2017. Exploring the effects of the “Grain for Green” program on the differences in soil water in the semi-arid Loess Plateau of China. Ecol. Eng. 107, 144–151. https://doi.org/10.1016/j.ecoleng.2017.07.017

Cao, S., 2011. Impact of China’s Large-Scale Ecological Restoration Program on the Environment and Society in Arid and Semiarid Areas of China: Achievements, Problems, Synthesis, and Applications. Crit. Rev. Environ. Sci. Technol. 4, 317–335. https://doi.org/10.1080/10643380902800034.

Cao, S., Xu, C., Chen, L., Wang, X., 2009. Attitudes of farmers in China’s northern Shaanxi Province towards the land-use changes required under the Grain for Green Project, and implications for the project’s success. Land Use Policy. 26, 1182–1194. https://doi.org/10.1016/j.landusepol.2009.02.006.

Dang, X., Gao, S., Tao, R., Liu, G., Xia, Z., Fan, L., Bi, W., 2020. Do environmental conservation programs contribute to sustainable livelihoods? Evidence from China’s grain-for-green program in northern Shaanxi province. Sci. Total Environ. 719, 137436. https://doi.org/10.1016/j.scitotenv.2020.137436.

Deng, L., Kim, D.G., Li, M., Huang, C., Liu, Q., Cheng, M., Peng, C., 2019. Land-use changes driven by “Grain for Green” program reduced carbon loss induced by soil erosion on the Loess Plateau of China. Glob. Planet. Change. 177, 101–115. https://doi.org/10.1016/j.gloplacha.2019.03.017.

Deng, L., Shangguan, Z., Sweeney, S., 2014. “Grain for Green” driven land use change and carbon sequestration on the Loess Plateau, China. Sci. Rep. 4. https://doi.org/10.1038/srep07039.

Deng, L., Wang, G., Liu, G., Shangguan, Z., 2016. Effects of age and land-use changes on soil carbon and nitrogen sequestrations following cropland abandonment on the Loess Plateau, China. Ecol. Eng. 90, 105–112. https://doi.org/10.1016/j.ecoleng.2016.01.086.

Dou, Y., Yang, Y., An, S., Zhu, Z., 2020. Effects of different vegetation restoration measures on soil aggregate stability and erodibility on the Loess Plateau, China. Catena. 185, 104294. https://doi.org/10.1016/j.catena.2019.104294.

Fan, X., Ma, Z., Yang, Q., Han, Y., Mahmood, R., Zheng, Z., 2014. Land use/land cover changes and regional climate over the Loess Plateau during 2001–2009. Part I: observational evidence. Clim. Change, 129, 427–440. https://doi.org/10.1007/s10584-014-1069-4.

Fang, L., Huimin, Y., Fengxue, G., Zhongen, N., Mei, H., 2017. Net primary productivity increased on the Loess Plateau following implementation of the grain to green program. JRE. 8, 413–421. https://doi.org/10.5814/j.issn.1674-764x.2017.04.014.

Feng, Q., Zhao, W., Fu, B., Ding, J., Wang, S., 2017. Ecosystem service trade-offs and their influencing factors: A case study in the Loess Plateau of China. Sci. Total Environ. 607-608, 1250 – 1263. https://doi.org/10.1016/j.scitotenv.2017.07.079.

Feng, X., Fu, B., Lu, N., Zeng, Y., Wu, B., 2013. How ecological restoration alters ecosystem services: an analysis of carbon sequestration in China’s Loess Plateau. Sci. Rep. 3. https://doi.org/10.1038/srep02846.

Feng, X., Fu, B., Piao, S., Wang, S., Ciais, P., Zeng, Z., Lü, Y., Zeng, Y., Li, Y., Jiang, X., Wu, B., 2016. Revegetation in China’s Loess Plateau is approaching sustainable water resource limits. Nat. Clim. Change. 6, 1019–1022. https://doi.org/10.1038/nclimate3092.

Feng, X.M., Sun, G., Fu, B.J., Su, C.H., Liu, Y., Lamparski, H., 2012. Regional effects of vegetation restoration on water yield across the Loess Plateau, China. Hydrol. Earth Syst. Sci. Discuss. 16, 2617–2628. https://doi.org/10.5194/hess-16-2617-2012.

Fu, B., Liu, Y., Lü, Y., He, C., Zeng, Y., Wu, B., 2011. Assessing the soil erosion control service of ecosystems change in the Loess Plateau of China. Ecol. Complex. 8, 284–293. https://doi.org/10.1016/j.ecocom.2011.07.003.

Fu, X., Shao, M., Wei, X., Horton, R., 2010. Soil organic carbon and total nitrogen as affected by vegetation types in Northern Loess Plateau of China. Geoderma. 155, 31–35. https://doi.org/10.1016/j.geoderma.2009.11.020.

Gang, C., Zhao, W., Zhao, T., Zhang, Y., Gao, X., Wen, Z., 2018. The impacts of land conversion and management measures on the grassland net primary productivity over the Loess Plateau, Northern China. Sci. Total Environ. 645, 827 – 836. https://doi.org/10.1016/j.scitotenv.2018.07.161.

Ge, J., Pitman, A.J., Guo, W., Zan, B., Fu, C., 2020. Impact of revegetation of the Loess Plateau of China on the regional growing season water balance. Hydrol. Earth Syst. Sci. 24, 515–533. https://doi.org/10.5194/hess-24-515-2020.

Geng, Q., Ren, Q., Yan, H., Li, L., Zhao, X., Mu, X., Wu, P., Yu, Q., 2019. Target areas for harmonizing the Grain for Green Programme in China’s Loess Plateau. Land Degrad. Dev. 31, 325–333. https://doi.org/10.1002/ldr.3451.

Gong, J., Chen, L., Fu, B., Huang, Y., Huang, Z., Peng, H., 2006. Effect of land use on soil nutrients in the loess hilly area of the Loess Plateau, China. Land Degrad. Dev. 17, 453–465. https://doi.org/10.1002/ldr.701.

Guo, B., Xie, T., Subrahmanyam, M.V., 2019. The Impact of China’s Grain for Green Program on Rural Economy and Precipitation: A Case Study of Yan River Basin in the Loess Plateau. Sustainability. 11, 5336. https://doi.org/10.3390/su11195336.

Guo, L., Di, L., Li, G., Luo, Q., Gao, M., 2015. GIS-based detection of land use transformation in the Loess Plateau: A case study in Baota District, Shaanxi Province, China. JJ. Geogr. Sci. 25, 1467–1478. https://doi.org/10.1007/s11442-015-1246-z.

Jiang, C., Zhang, H., Zhang, Z., 2018. Spatially explicit assessment of ecosystem services in China’s Loess Plateau: Patterns, interactions, drivers, and implications. Glob. Planet. Change. 161, 41–52. https://doi.org/10.1016/j.gloplacha.2017.11.014

Jiao, J., Zhang, Z., Bai, W., Jia, Y., Wang, N., 2010. Assessing the Ecological Success of Restoration by Afforestation on the Chinese Loess Plateau. Ecol. Restor. 2, 240–249. https://doi.org/10.1111/j.1526-100x.2010.00756.x.

Liu, C., Li, Z., Dong, Y., Nie, X., Liu, L., Xiao, H., Zeng, G., 2017. Do land use change and check-dam construction affect a real estimate of soil carbon and nitrogen stocks on the Loess Plateau of China? Ecol. Eng. 101, 220–226. https://doi.org/10.1016/j.ecoleng.2017.01.036

Lü, Y., Fu, B., Feng, X., Zeng, Y., Liu, Y., Chang, R., Sun, G., Wu, B., 2012. A Policy-driven large-scale ecological restoration: quantifying ecosystem services changes in the Loess Plateau of China. PLoS ONE 7. https://doi.org/10.1371/journal.pone.0031782.

Lv, M., Ma, Z., Li, M., Zheng, Z., 2019. Quantitative analysis of terrestrial water storage changes under the Grain for Green Program in the Yellow River Basin. J. Geophys. Res. Solid Earth. 124, 1336–1351. https://doi.org/10.1029/2018jd029113.

Lyu, C., Xu, Z., 2020. Crop production changes and the impact of Grain for Green program in the Loess Plateau of China. J. Arid Land Stud. 12, 18–28. https://doi.org/10.1007/s40333-020-0091-9.

Shi, P., Feng, Z., Gao, H., Li, P., Zhang, X., Zhu, T., Li, Z., Xu, G., Ren, Z., Xiao, L., 2020. Has “Grain for Green” threaten food security on the Loess Plateau of China? Ecosyst Health Manag. 6, 1709560. https://doi.org/10.1080/20964129.2019.1709560.

Su, B., & Shangguan, Z., 2018. Decline in soil moisture due to vegetation restoration on the Loess Plateau of China. Land Degrad Dev. 30, 290–299. https://doi.org/10.1002/ldr.3223.

Sun, W., Shao, Q., Liu, J., 2013. Soil erosion and its response to the changes of precipitation and vegetation cover on the Loess Plateau. J. Geogr. Sci. 23, 1091–1106. https://doi.org/10.1007/s11442-013-1065-z.

Tuo, D., Gao, G., Chang, R., Li, Z., Ma, Y., Wang, S., Wang, C., Fu, B., 2018. Effects of revegetation and precipitation gradient on soil carbon and nitrogen variations in deep profiles on the Loess Plateau of China. Sci. Total Environ. 626, 399 – 411. https://doi.org/10.1016/j.scitotenv.2018.01.116.

Wang, J., Liu, Y., Liu, Z., 2013. Spatio-Temporal Patterns of Cropland Conversion in Response to the “Grain for Green Project” in China’s Loess Hilly Region of Yanchuan County. Remote. Sens. 5, 5642 – 5661. https://doi.org/10.3390/rs5115642.

Wang, T., Kang, F., Cheng, X., Han, H., Ji, W., 2016. Soil organic carbon and total nitrogen stocks under different land uses in a hilly ecological restoration area of North China. Soil Tillage Res. 163, 176–184. https://doi.org/10.1016/j.still.2016.05.015

Wang, Y., Brandt, M., Zhao, M., Tong, X., Xing, K., Xue, F., Kang, M., Wang, L., Jiang, Y., Fensholt, R., 2018. Major forest increase on the Loess Plateau, China (2001–2016). Land Degrad. Dev. 29, 4080–4091. https://doi.org/10.1002/ldr.3174.

Wang, Y., Kang, M., Zhao, M., Xing, K., Wang, G., Xue, F., 2017. The Spatiotemporal Variation of Tree Cover in the Loess Plateau of China after the “Grain for Green” Project. Sustainability. 9, 739. https://doi.org/10.3390/su9050739.

Wei, H., Fan, W., Ding, Z., Weng, B., Xing, K., Wang, X., Lu, N., Ulgiati, S., Dong, X., 2017. Ecosystem Services and Ecological Restoration in the Northern Shaanxi Loess Plateau, China, in relation to Climate Fluctuation and Investments in Natural Capital. Sustainability. 9, 199. https://doi.org/10.3390/su9020199.

Wen, X., Zhen, L., 2020. Soil erosion control practices in the Chinese Loess Plateau: A systematic review. Environ. Dev. 34, 100493. https://doi.org/10.1016/j.envdev.2019.100493

Wu D, Zou C, Cao W, Xiao T, Gong G., 2019. Ecosystem services changes between 2000 and 2015 in the Loess Plateau, China: A response to ecological restoration. PLoS ONE. 14:e0209483. https://doi.org/10.1371/journal.pone.0209483.

Wu, X., Wang, S., Fu, B., Feng, X., Chen, Y., 2019. Socio-ecological changes on the Loess Plateau of China after Grain to Green Program. Sci. Total Environ. 678, 565 – 573. https://doi.org/10.1016/j.scitotenv.2019.05.022.

Xiao, J.-f., Xiao, J., 2014. Satellite evidence for significant biophysical consequences of the Grain for Green Program on the Loess Plateau in China. J. Geophys. Res. Solid Earth. 119, 2261–2275. https://doi.org/10.1002/2014jg002820.

Xu, Z., Bennett, M.T., Tao, R., Xu, J., 2004. China’s Sloping Land Conversion Program Four Years on: Current Situation and Pending Issues. Int. For. Rev. 6, 317–326. https://doi.org/10.1505/ifor.6.3.317.59976.

Yang, L., Chen, L., Wei, W., Yu, Y., Zhang, H., 2014. Comparison of deep soil moisture in two re-vegetation watersheds in semi-arid regions. J. Hydrol. 513, 314 – 321. https://doi.org/10.1016/j.jhydrol.2014.03.049.

Zhang, Y., Guo, S., Liu, Q., Jiang, J., Wang, R., Li, N., 2015. Responses of soil respiration to land use conversions in degraded ecosystem of the semi-arid Loess Plateau. Ecol. Eng. 74, 196–205. https://doi.org/10.1016/j.ecoleng.2014.10.003.

Zhao, A., Zhang, A., Liu, J., Feng, L., Zhao, Y., 2019. Assessing the effects of drought and “Grain for Green” Program on vegetation dynamics in China’s Loess Plateau from 2000 to 2014. Catena, 175, 446–455. https://doi.org/10.1016/j.catena.2019.01.013.

Zhou, H., Van Rompaey, A., Wang, J., 2009. Detecting the impact of the “Grain for Green” program on the mean annual vegetation cover in the Shaanxi province, China using SPOT-VGT NDVI data. Land Use Policy, 26(4), 954–960. https://doi.org/10.1016/j.landusepol.2008.11.006.
