# Supplementary material for: Ecological restoration stimulates environmental outcomes but exacerbates water shortage in the Loess Plateau
Source: PeerJ. 2022 Jul 8;10:e13658. doi: 10.7717/peerj.13658 (PMC9272815; doi:10.7717/peerj.13658)
Supplement: Supplemental Information 9 [file peerj-10-13658-s009.doc]

# Table S2. Ecosystem services assessment, data types and description of the selected articles.

| **Services assessment** | **Variables assessed** | **Methods/Parameters used** | **References** |
| --- | --- | --- | --- |
| Carbon sequestration | Net Primar Production (NPP) | Gross primary productivity less the costs associated with annual maintenance and growth respiration | [(Wu et al., 2019)](../../../../C:/Users/Junior%20Ngaba/AppData/Roaming/Microsoft/Word/Book4.xls#Sheet4!_ENREF_11) |
| Carnegie-Ames-Stanford Approach | [(Jiang et al., 2018)](../../../../C:/Users/Junior%20Ngaba/AppData/Roaming/Microsoft/Word/Book4.xls#Sheet4!_ENREF_6) |
| Carnegie Ames Stanford Approach (CASA) | [(Wei et al., 2017)](../../../../C:/Users/Junior%20Ngaba/AppData/Roaming/Microsoft/Word/Book4.xls#Sheet4!_ENREF_10) |
| Carbon sequestration (V*carbon*) and oxygen release (V*oxygen*) (economic value) | (Geng et al., 2020) |
| Gross primary productivity (GPP) | [(Wu et al., 2019)](../../../../C:/Users/Junior%20Ngaba/AppData/Roaming/Microsoft/Word/Book4.xls#Sheet4!_ENREF_12) |
| Carbon (SOC, concentrations and stocks) | Equations (C*_above_* and C*_below_* represent above-ground and underground biomass, C*_soil_* is soil carbon storage. C*_dead_* is the litter. | (Shi et al., 2020) |
| Equations (BD, SOC, soil thickness) | (Deng et al., 2018) |
| InVEST Carbon Storage and Sequestration model | [(Feng et al., 2020)](../../../../C:/Users/Junior%20Ngaba/AppData/Roaming/Microsoft/Word/Book4.xls#Sheet4!_ENREF_3) |
| Multiple regression approach | [(Lü et al., 2012)](../../../../C:/Users/Junior%20Ngaba/AppData/Roaming/Microsoft/Word/Book4.xls#Sheet4!_ENREF_8) |
| Equations (SOC, BD, D is the thickness) | (Deng et al., 2014) |
| Equations | (Liu et al., 2017) |
| Equations | (Tuo et al., 2018) |
| Equations | (Wang et al., 2016) |
| Soil respiration | Automated closed soil CO2 flux system | (Zhang et al., 2015) |
| Hydrological regulation | Water yield/regulation | Modeled as precipitation minus evapotranspiration | (Shi et al., 2020) |
| Modeled as precipitation minus evapotranspiration | (Fu et al., 2010) |
| Modeled as precipitation minus evapotranspiration | (Geng et al., 2020) |
| Modeled as precipitation minus evapotranspiration | [(Lü et al., 2012)](../../../../C:/Users/Junior%20Ngaba/AppData/Roaming/Microsoft/Word/Book4.xls#Sheet4!_ENREF_8) |
| Modeled as precipitation minus evapotranspiration | [(Wei et al., 2017)](../../../../C:/Users/Junior%20Ngaba/AppData/Roaming/Microsoft/Word/Book4.xls#Sheet4!_ENREF_10) |
| Annual evapotranspiration and annual precipitation | [(Feng et al., 2020)](../../../../C:/Users/Junior%20Ngaba/AppData/Roaming/Microsoft/Word/Book4.xls#Sheet4!_ENREF_3) |
| Potential evapotranspiration, precipitation and seasonal vegetation biomass dynamics (i.e., Leaf Area Index) | (Fu et al., 2010) |
| Rainfall storage method | [(Jiang et al., 2018)](../../../../C:/Users/Junior%20Ngaba/AppData/Roaming/Microsoft/Word/Book4.xls#Sheet4!_ENREF_6) |
| Precipitation storage method | [(Wu et al., 2019a)](../../../../C:/Users/Junior%20Ngaba/AppData/Roaming/Microsoft/Word/Book4.xls#Sheet4!_ENREF_11) |
| Runoff coefficient, MODIS ET data (Water supply service) | [(Wu et al., 2019)](../../../../C:/Users/Junior%20Ngaba/AppData/Roaming/Microsoft/Word/Book4.xls#Sheet4!_ENREF_12) |
| Soil water content | Number of measurement layers at site j, and θi is the mean soil moisture content in layers | (Yang et al., 2014) |
| Laboratory | [(Jiao et al., 2012)](../../../../C:/Users/Junior%20Ngaba/AppData/Roaming/Microsoft/Word/Book4.xls#Sheet4!_ENREF_11) |
| The fresh weight of the soil sample and the dry weight of the soil sample | (An et al., 2017) |
| Land use cover/change | Vegetation cover/change | Normalized Difference Vegetation Index (NDVI) | [(Jiang et al., 2018)](../../../../C:/Users/Junior%20Ngaba/AppData/Roaming/Microsoft/Word/Book4.xls#Sheet4!_ENREF_6) |
| Normalized Difference Vegetation Index (NDVI) | [(Wu et al., 2019a)](../../../../C:/Users/Junior%20Ngaba/AppData/Roaming/Microsoft/Word/Book4.xls#Sheet4!_ENREF_11) |
| Normalized Difference Vegetation Index (NDVI) | (Cao et al., 2019) |
| Normalized Difference Vegetation Index (NDVI) | (Fan et al., 2014) |
| Normalized Difference Vegetation Index (NDVI) | (Guo et al., 2015) |
| Normalized Difference Vegetation Index (NDVI) | (Wang et al., 2018) |
| Normalized Difference Vegetation Index (NDVI) | (Xu et al., 2020) |
| Normalized Difference Vegetation Index (NDVI) | (Zhao et al., 2019) |
| Normalized Difference Vegetation Index (NDVI) | (Sun et al., 2013) |
| Normalized Difference Vegetation Index (NDVI) | (Fu et al., 2011) |
| Normalized Difference Vegetation Index (NDVI) | (Ge et al., 2020) |
| Normalized Difference Vegetation Index (NDVI) | (Feng et al., 2016) |
| Normalized Difference Vegetation Index (NDVI) | [(Wu et al., 2019b)](../../../../C:/Users/Junior%20Ngaba/AppData/Roaming/Microsoft/Word/Book4.xls#Sheet4!_ENREF_12) |
| Normalized Difference Vegetation Index (NDVI) | (Ge et al., 2020) |
| Normalized Difference Vegetation Index (NDVI) | [(Guo et al., 2019)](../../../../C:/Users/Junior%20Ngaba/AppData/Roaming/Microsoft/Word/Book4.xls#Sheet4!_ENREF_5) |
| Normalized Difference Vegetation Index (NDVI) | (Zhou et al., 2009) |
| Remote sensing, Moderate Resolution Imaging Spectroradiometer (MODIS) | [(Wei et al., 2017)](../../../../C:/Users/Junior%20Ngaba/AppData/Roaming/Microsoft/Word/Book4.xls#Sheet4!_ENREF_10) |
| Carnegie-Ames-Stanford Approach (CASA) model | (Gang et al., 2018) |
| Remote sensing data, Landsat TM/ETM images | [(Lü et al., 2012)](../../../../C:/Users/Junior%20Ngaba/AppData/Roaming/Microsoft/Word/Book4.xls#Sheet4!_ENREF_8) |
| Slope Gradient of Converted Cropland | Wang et al. (2013) |
| Area of the entire study area; tree cover value of the year and the number of tree-covered | (Wang et al., 2017) |
| MODIS Vegetation Continuous Fields (VCF) product (MOD44B) | (Xiao and Xiao, 2014) |
| Net Primary Productivity | Normalized Difference Vegetation Index (NDVI) | (Fang et al., 2017) |
| (Deng et al., 2014) |
| Vegetation structure | Vegetation cover, height, DBH, canopy diameter, vertical stratification, and dominant species | [(Jiao et al., 2012)](../../../../C:/Users/Junior%20Ngaba/AppData/Roaming/Microsoft/Word/Book4.xls#Sheet4!_ENREF_7) |
| Plant diversity | Species richness, Shannon-Wiener index, Margalef index, Pielou index and Sorensen’s similarity index | (Jiao et al., 2012) |
| Soil conservation | Soil erosion/Loss | Revised Universal Soil Loss equation (RUSLE) | [(Jiang et al., 2018)](../../../../C:/Users/Junior%20Ngaba/AppData/Roaming/Microsoft/Word/Book4.xls#Sheet4!_ENREF_6) |
| (Sun et al., 2013) |
| [(Wu et al., 2019a)](../../../../C:/Users/Junior%20Ngaba/AppData/Roaming/Microsoft/Word/Book4.xls#Sheet4!_ENREF_11) |
| Universal Soil Loss Equation (USLE) | [(Lü et al., 2012)](../../../../C:/Users/Junior%20Ngaba/AppData/Roaming/Microsoft/Word/Book4.xls#Sheet4!_ENREF_8) |
| [(Wu et al., 2019 b)](../../../../C:/Users/Junior%20Ngaba/AppData/Roaming/Microsoft/Word/Book4.xls#Sheet4!_ENREF_12) |
| [(Feng et al., 2020)](../../../../C:/Users/Junior%20Ngaba/AppData/Roaming/Microsoft/Word/Book4.xls#Sheet4!_ENREF_11) |
| (Geng et al., 2020) |
| (Shi et al., 2020) |
| [(Fu et al., 2011)](../../../../C:/Users/Junior%20Ngaba/AppData/Roaming/Microsoft/Word/Book4.xls#Sheet4!_ENREF_11) |
| Literature review | [Wen et al. (2020)](../../../../C:/Users/Junior%20Ngaba/AppData/Roaming/Microsoft/Word/Book4.xls#Sheet4!_ENREF_11) |
| Soil aggregate | Water-stable soil aggregates | [(Jiao et al., 2012)](../../../../C:/Users/Junior%20Ngaba/AppData/Roaming/Microsoft/Word/Book4.xls#Sheet4!_ENREF_11) |
| Mean weight diameter (MWD) | [(Jiao et al., 2012)](../../../../C:/Users/Junior%20Ngaba/AppData/Roaming/Microsoft/Word/Book4.xls#Sheet4!_ENREF_11) |
| Equation | [(Dou et al., 2020)](../../../../C:/Users/Junior%20Ngaba/AppData/Roaming/Microsoft/Word/Book4.xls#Sheet4!_ENREF_11) |
| Modified Yoder’s method and Le Bissonnais method | [(An et al., 2013)](../../../../C:/Users/Junior%20Ngaba/AppData/Roaming/Microsoft/Word/Book4.xls#Sheet4!_ENREF_11) |
| Ratio of soil structure dispersion | / | [(Jiao et al., 2012)](../../../../C:/Users/Junior%20Ngaba/AppData/Roaming/Microsoft/Word/Book4.xls#Sheet4!_ENREF_11) |
| Soil nutrient levels | / | [(Jiao et al., 2012)](../../../../C:/Users/Junior%20Ngaba/AppData/Roaming/Microsoft/Word/Book4.xls#Sheet4!_ENREF_11) |
| Perception of repondants (Ecological success) | Interviews | [(Jiao et al., 2012)](../../../../C:/Users/Junior%20Ngaba/AppData/Roaming/Microsoft/Word/Book4.xls#Sheet4!_ENREF_11) |
| Sand fixation | Revised Wind Erosion Equation (RWEQ) | [(Wu et al., 2019a)](../../../../C:/Users/Junior%20Ngaba/AppData/Roaming/Microsoft/Word/Book4.xls#Sheet4!_ENREF_11) |
| Soil erodibility | Equation | (Dou et al., 2020) |
| Runoff C fluxes, Sediment C stock, Annual runoff coefficient, runoff and reduction, Sediment reduction, C loss reduction in runoff, C loss reduction in sediment | / | (Deng et al., 2019) |
| Trade-offs of Ess | / | The root mean squared error (RMSE) | [(Feng et al., 2020)](../../../../C:/Users/Junior%20Ngaba/AppData/Roaming/Microsoft/Word/Book4.xls#Sheet4!_ENREF_3) |

# References

An, W., Li, Z., Wang, S., Wu, X., Lu, Y., Liu, G., Fu, B., 2017. Exploring the effects of the “Grain for Green” program on the differences in soil water in the semi-arid Loess Plateau of China. Ecol. Eng. 107, 144–151. https://doi.org/10.1016/j.ecoleng.2017.07.017.

An, W., Li, Z., Wang, S., Wu, X., Lu, Y., Liu, G., Fu, B., 2017. Exploring the effects of the “Grain for Green” program on the differences in soil water in the semi-arid Loess Plateau of China. Ecol. Eng. 107, 144–151. https://doi.org/10.1016/j.ecoleng.2017.07.017

Cao, S., 2011. Impact of China’s Large-Scale Ecological Restoration Program on the Environment and Society in Arid and Semiarid Areas of China: Achievements, Problems, Synthesis, and Applications. Crit. Rev. Environ. Sci. Technol. 4, 317–335. https://doi.org/10.1080/10643380902800034.

Cao, S., Xu, C., Chen, L., Wang, X., 2009. Attitudes of farmers in China’s northern Shaanxi Province towards the land-use changes required under the Grain for Green Project, and implications for the project’s success. Land Use Policy. 26, 1182–1194. https://doi.org/10.1016/j.landusepol.2009.02.006.

Dang, X., Gao, S., Tao, R., Liu, G., Xia, Z., Fan, L., Bi, W., 2020. Do environmental conservation programs contribute to sustainable livelihoods? Evidence from China’s grain-for-green program in northern Shaanxi province. Sci. Total Environ. 719, 137436. https://doi.org/10.1016/j.scitotenv.2020.137436.

Deng, L., Kim, D.G., Li, M., Huang, C., Liu, Q., Cheng, M., Peng, C., 2019. Land-use changes driven by “Grain for Green” program reduced carbon loss induced by soil erosion on the Loess Plateau of China. Glob. Planet. Change. 177, 101–115. https://doi.org/10.1016/j.gloplacha.2019.03.017.

Deng, L., Shangguan, Z., Sweeney, S., 2014. “Grain for Green” driven land use change and carbon sequestration on the Loess Plateau, China. Sci. Rep. 4. https://doi.org/10.1038/srep07039.

Deng, L., Wang, G., Liu, G., Shangguan, Z., 2016. Effects of age and land-use changes on soil carbon and nitrogen sequestrations following cropland abandonment on the Loess Plateau, China. Ecol. Eng. 90, 105–112. https://doi.org/10.1016/j.ecoleng.2016.01.086.

Dou, Y., Yang, Y., An, S., Zhu, Z., 2020. Effects of different vegetation restoration measures on soil aggregate stability and erodibility on the Loess Plateau, China. Catena. 185, 104294. https://doi.org/10.1016/j.catena.2019.104294.

Fan, X., Ma, Z., Yang, Q., Han, Y., Mahmood, R., Zheng, Z., 2014. Land use/land cover changes and regional climate over the Loess Plateau during 2001–2009. Part I: observational evidence. Clim. Change, 129, 427–440. https://doi.org/10.1007/s10584-014-1069-4.

Fang, L., Huimin, Y., Fengxue, G., Zhongen, N., Mei, H., 2017. Net primary productivity increased on the Loess Plateau following implementation of the grain to green program. JRE. 8, 413–421. https://doi.org/10.5814/j.issn.1674-764x.2017.04.014.

Feng, Q., Zhao, W., Fu, B., Ding, J., Wang, S., 2017. Ecosystem service trade-offs and their influencing factors: A case study in the Loess Plateau of China. Sci. Total Environ. 607-608, 1250 – 1263. https://doi.org/10.1016/j.scitotenv.2017.07.079.

Feng, X., Fu, B., Lu, N., Zeng, Y., Wu, B., 2013. How ecological restoration alters ecosystem services: an analysis of carbon sequestration in China’s Loess Plateau. Sci. Rep. 3. https://doi.org/10.1038/srep02846.

Feng, X., Fu, B., Piao, S., Wang, S., Ciais, P., Zeng, Z., Lü, Y., Zeng, Y., Li, Y., Jiang, X., Wu, B., 2016. Revegetation in China’s Loess Plateau is approaching sustainable water resource limits. Nat. Clim. Change. 6, 1019–1022. https://doi.org/10.1038/nclimate3092.

Feng, X.M., Sun, G., Fu, B.J., Su, C.H., Liu, Y., Lamparski, H., 2012. Regional effects of vegetation restoration on water yield across the Loess Plateau, China. Hydrol. Earth Syst. Sci. Discuss. 16, 2617–2628. https://doi.org/10.5194/hess-16-2617-2012.

Fu, B., Liu, Y., Lü, Y., He, C., Zeng, Y., Wu, B., 2011. Assessing the soil erosion control service of ecosystems change in the Loess Plateau of China. Ecol. Complex. 8, 284–293. https://doi.org/10.1016/j.ecocom.2011.07.003.

Fu, X., Shao, M., Wei, X., Horton, R., 2010. Soil organic carbon and total nitrogen as affected by vegetation types in Northern Loess Plateau of China. Geoderma. 155, 31–35. https://doi.org/10.1016/j.geoderma.2009.11.020.

Gang, C., Zhao, W., Zhao, T., Zhang, Y., Gao, X., Wen, Z., 2018. The impacts of land conversion and management measures on the grassland net primary productivity over the Loess Plateau, Northern China. Sci. Total Environ. 645, 827 – 836. https://doi.org/10.1016/j.scitotenv.2018.07.161.

Ge, J., Pitman, A.J., Guo, W., Zan, B., Fu, C., 2020. Impact of revegetation of the Loess Plateau of China on the regional growing season water balance. Hydrol. Earth Syst. Sci. 24, 515–533. https://doi.org/10.5194/hess-24-515-2020.

Geng, Q., Ren, Q., Yan, H., Li, L., Zhao, X., Mu, X., Wu, P., Yu, Q., 2019. Target areas for harmonizing the Grain for Green Programme in China’s Loess Plateau. Land Degrad. Dev. 31, 325–333. https://doi.org/10.1002/ldr.3451.

Gong, J., Chen, L., Fu, B., Huang, Y., Huang, Z., Peng, H., 2006. Effect of land use on soil nutrients in the loess hilly area of the Loess Plateau, China. Land Degrad. Dev. 17, 453–465. https://doi.org/10.1002/ldr.701.

Guo, B., Xie, T., Subrahmanyam, M.V., 2019. The Impact of China’s Grain for Green Program on Rural Economy and Precipitation: A Case Study of Yan River Basin in the Loess Plateau. Sustainability. 11, 5336. https://doi.org/10.3390/su11195336.

Guo, L., Di, L., Li, G., Luo, Q., Gao, M., 2015. GIS-based detection of land use transformation in the Loess Plateau: A case study in Baota District, Shaanxi Province, China. JJ. Geogr. Sci. 25, 1467–1478. https://doi.org/10.1007/s11442-015-1246-z.

Jiang, C., Zhang, H., Zhang, Z., 2018. Spatially explicit assessment of ecosystem services in China’s Loess Plateau: Patterns, interactions, drivers, and implications. Glob. Planet. Change. 161, 41–52. https://doi.org/10.1016/j.gloplacha.2017.11.014

Jiao, J., Zhang, Z., Bai, W., Jia, Y., Wang, N., 2010. Assessing the Ecological Success of Restoration by Afforestation on the Chinese Loess Plateau. Ecol. Restor. 2, 240–249. https://doi.org/10.1111/j.1526-100x.2010.00756.x.

Liu, C., Li, Z., Dong, Y., Nie, X., Liu, L., Xiao, H., Zeng, G., 2017. Do land use change and check-dam construction affect a real estimate of soil carbon and nitrogen stocks on the Loess Plateau of China? Ecol. Eng. 101, 220–226. https://doi.org/10.1016/j.ecoleng.2017.01.036

Lü, Y., Fu, B., Feng, X., Zeng, Y., Liu, Y., Chang, R., Sun, G., Wu, B., 2012. A Policy-driven large-scale ecological restoration: quantifying ecosystem services changes in the Loess Plateau of China. PLoS ONE 7. https://doi.org/10.1371/journal.pone.0031782.

Lv, M., Ma, Z., Li, M., Zheng, Z., 2019. Quantitative analysis of terrestrial water storage changes under the Grain for Green Program in the Yellow River Basin. J. Geophys. Res. Solid Earth. 124, 1336–1351. https://doi.org/10.1029/2018jd029113.

Lyu, C., Xu, Z., 2020. Crop production changes and the impact of Grain for Green program in the Loess Plateau of China. J. Arid Land Stud. 12, 18–28. https://doi.org/10.1007/s40333-020-0091-9.

Shi, P., Feng, Z., Gao, H., Li, P., Zhang, X., Zhu, T., Li, Z., Xu, G., Ren, Z., Xiao, L., 2020. Has “Grain for Green” threaten food security on the Loess Plateau of China? Ecosyst Health Manag. 6, 1709560. https://doi.org/10.1080/20964129.2019.1709560.

Su, B., & Shangguan, Z., 2018. Decline in soil moisture due to vegetation restoration on the Loess Plateau of China. Land Degrad Dev. 30, 290–299. https://doi.org/10.1002/ldr.3223.

Sun, W., Shao, Q., Liu, J., 2013. Soil erosion and its response to the changes of precipitation and vegetation cover on the Loess Plateau. J. Geogr. Sci. 23, 1091–1106. https://doi.org/10.1007/s11442-013-1065-z.

Tuo, D., Gao, G., Chang, R., Li, Z., Ma, Y., Wang, S., Wang, C., Fu, B., 2018. Effects of revegetation and precipitation gradient on soil carbon and nitrogen variations in deep profiles on the Loess Plateau of China. Sci. Total Environ. 626, 399 – 411. https://doi.org/10.1016/j.scitotenv.2018.01.116.

Wang, J., Liu, Y., Liu, Z., 2013. Spatio-Temporal Patterns of Cropland Conversion in Response to the “Grain for Green Project” in China’s Loess Hilly Region of Yanchuan County. Remote. Sens. 5, 5642 – 5661. https://doi.org/10.3390/rs5115642.

Wang, T., Kang, F., Cheng, X., Han, H., Ji, W., 2016. Soil organic carbon and total nitrogen stocks under different land uses in a hilly ecological restoration area of North China. Soil Tillage Res. 163, 176–184. https://doi.org/10.1016/j.still.2016.05.015

Wang, Y., Brandt, M., Zhao, M., Tong, X., Xing, K., Xue, F., Kang, M., Wang, L., Jiang, Y., Fensholt, R., 2018. Major forest increase on the Loess Plateau, China (2001–2016). Land Degrad. Dev. 29, 4080–4091. https://doi.org/10.1002/ldr.3174.

Wang, Y., Kang, M., Zhao, M., Xing, K., Wang, G., Xue, F., 2017. The Spatiotemporal Variation of Tree Cover in the Loess Plateau of China after the “Grain for Green” Project. Sustainability. 9, 739. https://doi.org/10.3390/su9050739.

Wei, H., Fan, W., Ding, Z., Weng, B., Xing, K., Wang, X., Lu, N., Ulgiati, S., Dong, X., 2017. Ecosystem Services and Ecological Restoration in the Northern Shaanxi Loess Plateau, China, in relation to Climate Fluctuation and Investments in Natural Capital. Sustainability. 9, 199. https://doi.org/10.3390/su9020199.

Wen, X., Zhen, L., 2020. Soil erosion control practices in the Chinese Loess Plateau: A systematic review. Environ. Dev. 34, 100493. https://doi.org/10.1016/j.envdev.2019.100493

Wu D, Zou C, Cao W, Xiao T, Gong G., 2019. Ecosystem services changes between 2000 and 2015 in the Loess Plateau, China: A response to ecological restoration. PLoS ONE. 14:e0209483. https://doi.org/10.1371/journal.pone.0209483.

Wu, X., Wang, S., Fu, B., Feng, X., Chen, Y., 2019. Socio-ecological changes on the Loess Plateau of China after Grain to Green Program. Sci. Total Environ. 678, 565 – 573. https://doi.org/10.1016/j.scitotenv.2019.05.022.

Xiao, J.-f., Xiao, J., 2014. Satellite evidence for significant biophysical consequences of the Grain for Green Program on the Loess Plateau in China. J. Geophys. Res. Solid Earth. 119, 2261–2275. https://doi.org/10.1002/2014jg002820.

Xu, Z., Bennett, M.T., Tao, R., Xu, J., 2004. China’s Sloping Land Conversion Program Four Years on: Current Situation and Pending Issues. Int. For. Rev. 6, 317–326. https://doi.org/10.1505/ifor.6.3.317.59976.

Yang, L., Chen, L., Wei, W., Yu, Y., Zhang, H., 2014. Comparison of deep soil moisture in two re-vegetation watersheds in semi-arid regions. J. Hydrol. 513, 314 – 321. https://doi.org/10.1016/j.jhydrol.2014.03.049.

Zhang, Y., Guo, S., Liu, Q., Jiang, J., Wang, R., Li, N., 2015. Responses of soil respiration to land use conversions in degraded ecosystem of the semi-arid Loess Plateau. Ecol. Eng. 74, 196–205. https://doi.org/10.1016/j.ecoleng.2014.10.003.

Zhao, A., Zhang, A., Liu, J., Feng, L., Zhao, Y., 2019. Assessing the effects of drought and “Grain for Green” Program on vegetation dynamics in China’s Loess Plateau from 2000 to 2014. Catena, 175, 446–455. https://doi.org/10.1016/j.catena.2019.01.013.

Zhou, H., Van Rompaey, A., Wang, J., 2009. Detecting the impact of the “Grain for Green” program on the mean annual vegetation cover in the Shaanxi province, China using SPOT-VGT NDVI data. Land Use Policy, 26(4), 954–960. https://doi.org/10.1016/j.landusepol.2008.11.006.
